# General protocol for predicting outbreaks of infectious diseases in social networks

**DOI:** 10.1038/s41598-024-56340-7

**Published:** 2024-03-12

**Authors:** Sungchul Kwon, Jeong-Man Park

**Affiliations:** Department of Physics, The Catholic University of Korea, Bucheon, 14662 Korea

**Keywords:** Biophysics, Mathematics and computing, Physics

## Abstract

Epidemic spreading on social networks with quenched connections is strongly influenced by dynamic correlations between connected nodes, posing theoretical challenges in predicting outbreaks of infectious diseases. The quenched connections introduce dynamic correlations, indicating that the infection of one node increases the likelihood of infection among its neighboring nodes. These dynamic correlations pose significant difficulties in developing comprehensive theories for threshold determination. Determining the precise epidemic threshold is pivotal for diseases control. In this study, we propose a general protocol for accurately determining epidemic thresholds by introducing a new set of fundamental conditions, where the number of connections between individuals of each type remains constant in the stationary state, and by devising a rescaling method for infection rates. Our general protocol is applicable to diverse epidemic models, regardless of the number of stages and transmission modes. To validate our protocol’s effectiveness, we apply it to two widely recognized standard models, the susceptible–infected–recovered-susceptible model and the contact process model, both of which have eluded precise threshold determination using existing sophisticated theories. Our results offer essential tools to enhance disease control strategies and preparedness in an ever-evolving landscape of infectious diseases.

Networks are common structures observed in both real and artificial worlds, encompassing brain neurons, food chains, public transport, the World Wide Web, and social relationships. Mathematically, a network is described as a graph comprising nodes connected by links. The number of links of a node is referred to as its degree, denoted as *k*. *P*(*k*) represents the degree distribution, which is usually Poissonian or power-law. Networks are categorized as either annealed networks with time-varying connections or quenched networks with fixed connections over time^1–4^. We focus on quenched networks.

Epidemic spreading is one of the most important dynamical processes taking place on networks. The primary objectives of studying epidemic spreading are to understand the mechanisms of infectious diseases’ dissemination and, importantly, to predict epidemic outbreaks. For these purposes, modeling epidemic spreading and finding the accurate thresholds of various standard models on complex networks have grown into an active research field and also led to the development of a diverse range of theoretical approaches taking into account the effects of heterogeneous connection patterns on the spreading of diseases^1–5^.

The heterogeneous connection pattern of underlying social network makes a significant impact on the spreading of epidemic and leads to novel phenomena that go beyond the scope of ordinary mean-field theories^3,4^. In the case of quenched networks, susceptible individuals having connections with infected ones are likely to be infected, which is referred to the dynamic correlation and crucial in determining the thresholds^3,4^. The existence of these dynamic correlations represents a longstanding challenge that remains unresolved in the development of comprehensive methods for accurately determining thresholds in diverse epidemic models with multiple stages of disease progression and various transmission modes on quenched networks. This necessitates the exploration of fundamental principles underlying a variety of spreading dynamics on quenched networks.

The aim of the present study is to provide a general procedure for deriving accurate epidemic thresholds by introducing a new set of fundamental conditions satisfied in the stationary state of various spreading dynamics and devising a rescaling method for infection rates. Our protocol is applicable to diverse epidemic models with multiple stages and various ways of transmission on a wide range of quenched networks.

In epidemic models, the population is typically divided into different compartments based on the stage of the disease, such as susceptible (S), infectious (I), and recovered (R)^5–7^. Hence various models have been introduced according to the number of the stage of diseases under consideration. The simplest model is the susceptible–infected (SI) model, which assumes that the disease leads to lifelong infections without recovery, undergoing an irreversible flow $S \overset{\lambda }{\rightarrow }\ I$, where $\lambda$ represents the infection rate. The susceptible–infected–susceptible (SIS) model assumes that the disease does not grant immunity, causing individuals to be infected and recovered without immunity, undergoing a cycle $S \overset{\lambda }{\rightarrow }\ I \overset{r}{\rightarrow }\ S$, with *r* representing the recovery rate. The susceptible–infected–recovered (SIR) model, in contrast, considers infected individuals to gain permanent immunity after recovery, undergoing an irreversible flow $S \overset{\lambda }{\rightarrow }\ I \overset{r}{\rightarrow }\ R$.

However, in the case of many infectious diseases such as influenza and the recent COVID-19 pandemic, immunity and infection are not lifelong. In reality, the more relevant model is the susceptible–infected–recovered–susceptible (SIRS) model, where an infected individual acquires temporary immunity upon recovery, undergoing a cycle $S \overset{\lambda }{\rightarrow }\ I \overset{r}{\rightarrow }\ R \overset{h}{\rightarrow }\ S$, with *h* representing the loss-of-immunity rate. The SIRS model is highly comprehensive, as it encompasses the SI, SIR, and SIS models as special cases, obtained by taking the limits $r \rightarrow 0$, $h \rightarrow 0$, and $h \rightarrow \infty$, respectively. Note that all models mentioned so far adopt the transmission way that all susceptible nodes connected to an infected node are infected at the same time with the rate $\lambda$.

The SIS model is the prototype of epidemic models^3,4,8–17^. In the thermodynamic limit of an infinite population, the SIS model experiences an absorbing phase transition (APT) between the disease-free (absorbing) phase and the endemic (active) phase when the parameter $\bar{\lambda } \equiv \lambda /r$ is tuned^18–20^. The epidemic threshold $\bar{\lambda }_{c}$ is the $\bar{\lambda }$ value at which this APT occurs.

Numerous theoretical endeavors have been dedicated to determining the epidemic thresholds for both the SIS and SIR models on quenched networks^1–5,8–15^. The heterogeneous mean-field (HMF) theory has been applied to the study of both models^4,8,9^ and predicted that the epidemic thresholds of the SIS and SIR models are the same, given by $\bar{\lambda }_{c}^{\text{HMF}} = \langle k \rangle /\langle k^{2} \rangle$^4,25–27^. The symbols $\langle k \rangle$ and $\langle k^{2} \rangle$ are the first and the second moments of degree *k*. However, several numerical studies have indicated that simulation results for epidemic thresholds deviate from the threshold values predicted by the HMF theory^27–29^.

For the SIR model, mapping the SIR model to a bond-percolation problem provided a notably more accurate prediction of the epidemic threshold for the SIR model on quenched networks, given by $\bar{\lambda }_{c}^{\text{SIR}} = [\langle k \rangle /(\langle k^{2} \rangle - 2 \langle k \rangle )]$^4,30,31^. For the SIS model, Cai *et al.* incorporated dynamic correlations into a combined theory by integrating the HMF theory with the effective degree (ED) approach and predicted the epidemic threshold to be $\bar{\lambda }_{c}^{\text{SIS}} = [\langle k \rangle /(\langle k^{2} \rangle - \langle k \rangle )]$^32^. Unfortunately, for the SIRS model, the combined theory by Cai *et al.* does not provide a complete set of equations necessary for the derivation of threshold and hence cannot be applied to models with more than two stages.

On the other hand, the contact process (CP) is a typical example of another transmission scheme different from the SIS and SIRS models. The CP also holds a prominent position as the archetype for APTs in lattices^19,20^. It shares similarities with SIS dynamics, with the key distinction lying in the fact that infection is attempted to a randomly selected susceptible neighbor only, rather than all susceptible neighbors, of the infected node. Similarly to the SIRS model, the accurate threshold of the CP on quenched networks remains elusive, despite the availability of results from the HMF theory and the self-consistent equation for the threshold through the heterogeneous pair-approximation^34,35^. The challenge here is the random neighbor selection, which poses a theoretical barrier to the application of the approach of Cai *et al.* to the CP.

Consequently, the current comprehension of the CP and SIRS dynamics on quenched networks remains at the HMF theory level^4,33^, which shows the importance of the number of disease stages and the way of transmission in addition to the importance of the dynamic correlations, and calls for a deeper theoretical understanding of the epidemic spreading on quenched networks.

In this work, we introduce a new set of fundamental conditions named the bond-detailed-balance (BDB) conditions, which the numbers of every type of two connected nodes (bonds) must satisfy in the stationary state, and a systematic way of rescaling infection rate to apply the new conditions. By integrating the BDB conditions and the rescaling method with the method of Cai *et al.*, we develop a general protocol (a series of the derivation procedures of thresholds), which can be applicable to diverse epidemic models with multiple numbers of disease stages and various transmission ways on quenched networks with an arbitrary *P*(*k*). As an example, the protocol applied to the SIRS model is depicted schematically in Fig. 1. We verify this protocol by deriving the accurate thresholds of the CP and SIRS model. The BDB conditions and the predicted thresholds of both models undergo meticulous numerical validation through Monte Carlo simulations conducted on quenched scale-free networks with $P(k) \sim k^{-\gamma }$ with $\gamma >2$.

**Figure 1:**
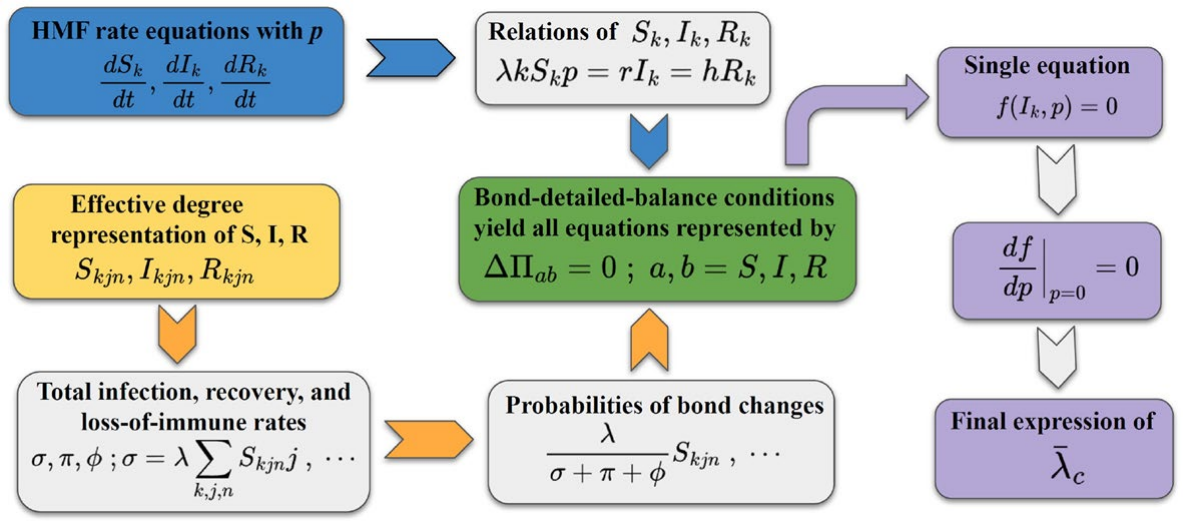
The protocol for the SIRS model to derive the threshold $\bar{\lambda }^{\text{SIRS}}_c$. The derivation procedure follows arrows. The yellow arrow indicates that inputs are utilized to obtain equations corresponding to bond-detailed-balance conditions for every type of bonds in terms of the effective-degree expressions of *S*, *I*, and *R*. Inputs entered along the blue arrow are utilized to express the obtained equations in terms of $I_k$ and *p*. The purple arrow indicates that all equations reduced to a single equation after some algebra. For the CP with another transmission way, the infection rate is first rescaled with $\langle k\rangle /\langle k^2 \rangle$ to make the HMF rate equation of $I_k$ similar to $dI_k /dt$ of the SIRS model before applying the protocol for the CP, which is obtained by dropping all variables related *R* in the protocol for the SIRS model.

## Results

### SIRS model

The derivation of the threshold of the SIRS model follows the protocol illustrated in Fig. 1.

We begin with employing the HMF approximation to establish the rate equations for the SIRS model on a quenched network characterized by an arbitrary *P*(*k*) distribution. Within the HMF approximation, nodes are categorized based on their degrees^8,9^. For a given degree *k*, we define the set $\Omega _{k}$ comprising nodes with the same degree, and denote $N_{k}$ as its size. We further define $S_{k} = \sum _{i\in \Omega _{k}} S_{i}$ as the count of nodes in the state *S* within the set $\Omega _{k}$. Similarly, $I_{k}$ and $R_{k}$ are defined for states *I* and *R*. Consequently, the total number of nodes with degree *k* is determined by $N_{k} = S_{k} + I_{k} + R_{k}$. By summing $S_{k}$, $I_{k}$, and $R_{k}$ across all possible *k* values, we obtain the overall numbers of nodes in states *S*, *I*, and *R*, represented as $S = \sum _{k=k_{\text{min}}}^{k_{\text{max}}} S_{k}$. Here, $k_{\text{min}}$ and $k_{\text{max}}$ correspond to the minimum and maximum degrees within the underlying network.

To include the dynamic correlations, Cai *et al.* combined the HMF theory with the ED approach that introduces the correlations between susceptible (infected) nodes and their infected neighbors by defining $p_{k}$ ($q_{k}$) as the probability of a susceptible (infected) node with degree *k* having an infected neighbor along a randomly chosen link among their *k* links^32^. With the ED approach, the HMF rate equations of $S_k$, $I_k$, and $R_k$ are modified as follows

1 $$\begin{aligned} \frac{dS_{k}}{dt}= & {} - \lambda k S_{k}p_{k} + h R_{k}, \end{aligned}$$

2 $$\begin{aligned} \frac{dI_{k}}{dt}= & {} -r I_{k} + \lambda k S_{k}p_{k}, \end{aligned}$$

3 $$\begin{aligned} \frac{dR_{k}}{dt}= & {} -h R_{k} + r I_{k} . \end{aligned}$$

In the steady state, $S_k$, $I_k$, and $R_k$ are related with each other by Eqs. (1)–(3) as

4 $$\begin{aligned} \lambda k S_{k}p_{k} = r I_{k} = h R_{k} . \end{aligned}$$

Similarly to the SIS model^32^, we introduce $S_{kjn}$, $I_{kjn}$, and $R_{kjn}$, denoting the numbers of nodes among $S_k$, $I_k$, and $R_k$ categories, which are connected to *j* infected neighbors and *n* recovered neighbors. For $S_{kjn}$, $I_{kjn}$, and $R_{kjn}$, we define the probabilities $p_{k}$, $q_{k}$, and $v_{k}$, which signify the likelihood of a susceptible node, an infected node, and a recovered node with degree *k*, respectively, being connected to an infected neighbor through a randomly selected link among *k* links. Additionally, we introduce $w_{k}$, $x_{k}$, and $y_{k}$ for $S_{kjn}$, $I_{kjn}$, and $R_{kjn}$, representing the probabilities of a susceptible node, an infected node, and a recovered node with degree *k* being connected to a recovered neighbor through a randomly chosen link among *k* links, respectively.

Within the set $\Omega _k$, we focus on nodes having *j* infected neighbors and *n* recovered neighbors out of *k* links. For a specific combination of *j* and *n*, we can express $S_{kjn}$, $I_{kjn}$, and $R_{kjn}$ using binomial distributions involving $p_{k}$, $q_{k}$, and $v_{k}$ for *j* infected neighbors, as well as $w_{k}$, $x_{k}$, and $y_{k}$ for *n* recovered neighbors

5 $$\begin{aligned} S_{kjn} = S_{k} (1-p_{k} )^{k-j} p_{k}^{j} \left( {\begin{array}{c}k\\ j\end{array}}\right) (1-\hat{w}_{k} )^{k-j-n}\hat{w}_{k}^{n} \left( {\begin{array}{c}k-j\\ n\end{array}}\right) , \end{aligned}$$

where $$\left( {\begin{array}{c}k\\ j\end{array}}\right) $$ represents a combination of *k* links taken *j* at a time without repetition and $\hat{w}_{k} = w_{k}/(1-p_{k})$ denotes the conditional probabilities of a link being connected to a recovered neighbor given that the link is not connected to an infected neighbor. $I_{kjn}$ and $R_{kjn}$ can be expressed by using binomial distributions similarly. Subsequently, $S_{k}$, $I_{k}$, and $R_{k}$ are calculated by summing $S_{kjn}$, $I_{kjn}$, and $R_{kjn}$ across the range of *j* and *n*. For further details, refer to the Supplemental Material^36^.

We define a bond as two nodes directly connected and present a new set of conditions that bonds must satisfy for epidemic dynamics on networks. We assert that the number ($\Pi _{ab}$) of each bond type remains constant on average in the steady state, where *a* and *b* can be any of *S*, *I*, and *R*. Consequently, the average change ($\langle \Delta \Pi _{ab}\rangle$) in the number of each bond type vanishes in a time interval *dt*, during which only a single event occurs. We refer to this as the bond-detailed-balance (BDB) condition.

In Table 1, we provide a summary of all possible changes in the numbers of various bond types, along with their corresponding probabilities. Using Table 1, we derive six equations that represent the BDB conditions by summing the product of each contribution to $\Delta \Pi _{ab}$ and the corresponding probability. We confirmed the validity of the BDB conditions through numerical simulations and provide comprehensive details of the simulation outcomes in the Supplemental Material. Among these six equations^36^, the following three equations are utilized:

6 $$\begin{aligned}{} & {} \langle \Delta \Pi _{II} \rangle = \sum _{kjn}\lambda S_{kjn}j \cdot j - \sum _{kjn} r I_{kjn} \cdot j = 0, \end{aligned}$$

7 $$\begin{aligned} \langle \Delta \Pi _{SI} \rangle =& \sum _{kjn}\lambda S_{kjn}j \cdot \left( (k-j-n) - j \right)\\ &- \sum _{kjn} r I_{kjn} \cdot (k-j-n) + \sum _{kjn} h R_{kjn} \cdot j = 0, \end{aligned}$$

8 $$\begin{aligned}\langle \Delta \Pi _{IR} \rangle = &\sum _{kjn}\lambda S_{kjn}j \cdot n \\ & + \sum _{kjn} r I_{kjn} \cdot (j-n) - \sum _{kjn} h R_{kjn} \cdot j = 0. \end{aligned}$$

**Table 1:** The changes in the numbers of bonds due to all possible events during a time interval *dt* of the SIRS model on a quenched network. $\Sigma$ represents the total rate $\Sigma = \sigma + \pi + \phi$ defined in the Supplemental Material^36^.

Event	$S_{kjn} \rightarrow I_{kjn}$	$I_{kjn} \rightarrow R_{kjn}$	$R_{kjn} \rightarrow S_{kjn}$
Probability	$\lambda S_{kjn} j/\Sigma$	$r I_{kjn}/\Sigma$	$h R_{kjn}/\Sigma$
$\Delta \Pi _{SS}$	$-(k-j-n)$	0	$(k-j-n)$
$\Delta \Pi _{II}$	*j*	$-j$	0
$\Delta \Pi _{RR}$	0	*n*	$-n$
$\Delta \Pi _{SI}$	$-j+(k-j-n)$	$-(k-j-n)$	*j*
$\Delta \Pi _{IR}$	*n*	$(j-n)$	$-j$
$\Delta \Pi _{RS}$	$-n$	$(k-j-n)$	$n-(k-j-n)$

By summing Eqs. (7) and (8) and utilizing Eq. (6), we deduce the relationship $\sum _{kjn}\lambda S_{kjn}jk = \sum _{kjn} r I_{kjn} k$ so that Eqs. (7) and (8) become equivalent and can be organized into the following form:

9 $$\begin{aligned} (r+h) \sum _{kjn} I_{kjn} n - \lambda \sum _{kjn} S_{kjn}j^{2} - \lambda \sum _{kjn} S_{kjn}jn = 0. \end{aligned}$$

By expressing the second and third terms using $I_{k}$^36^, Eq. (9) is rewritten as:

10 $$\begin{aligned}{} & {} ( r+h ) \left( \sum _{k} k I_{k} - \frac{r}{\lambda } \sum _{k} I_{k} \right) \nonumber \\{} & {} \quad -(2r+h)\sum _{k} \left( 1 + p_{k}(k-1) \right) I_{k} - r \sum _{k} w_{k} (k -1) I_{k} = 0 . \end{aligned}$$

Considering that we lack information about how $p_{k}$ varies for different *k* values, we assume, following the maximum entropy principle, that all $p_{k}$ and $w_{k}$ are the same across all *k* values ($p_{k} = p, w_{k} = w$ for all *k*).

Utilizing the relation from Eq. (4) and $S_k + I_k + R_k = N_k$ with $N_k = NP(k)$, we can express $I_k$ as a function of *p*:

11 $$\begin{aligned} I_k = \frac{\lambda kp N}{r + (1+r/h)\lambda kp} P(k) . \end{aligned}$$

Substituting Eq. (11) into Eq. (10), we arrive at a self-consistent equation for *p*. By finding the solution of *p* that satisfies this self-consistent equation and then substituting this solution back into Eq. (11), the total number of infected nodes in the stationary state can be determined^32^.

The epidemic threshold $\lambda _{c}$ can be determined from Eq. (10)^32^. We define the left-hand side of Eq. (10) as *f*(*p*). The trivial solution of $f(p)=0$ is $p=0$, corresponding to the disease-free phase due to $I_{k}|_{p=0}=0 \; \forall \; k$ from Eq. (11). Conversely, $f(p=1)$ is always negative. Hence, for a positive solution $p^+$ in the interval (0, 1) that corresponds to the endemic phase with positive $I(p^{+})$, *f*(*p*) must be a convex function of *p*. The convexity requirement implies that the slope of *f*(*p*) at $p=0$, $df(p)/dp |_{p=0}$, should be positive. As the solution $p^{+}$ and $I(p^{+})$ tend to zero with the control parameter $\lambda \rightarrow \lambda ^{+}_{c}$, the slope also approaches zero as $\lambda \rightarrow \lambda ^{+}_{c}$ and eventually becomes zero at $\lambda _{c}$. Hence, the condition for the transition between the disease-free and endemic phases is $\frac{df(p)}{dp} \bigg |_{p=0} = 0$, and we derive the epidemic threshold $\bar{\lambda }_{c} = \lambda _{c}/r$ of the SIRS model^36^ as

12 $$\begin{aligned} \bar{\lambda }_{c}^{\text{SIRS}} = \frac{\langle k \rangle }{\langle k^2 \rangle - ( \frac{h+2r}{h+r} ) \langle k \rangle } . \end{aligned}$$

From Eq. (12), we can immediately derive the threshold for the SIRS model on quenched regular networks with $P(k)=\delta _{k,k_{0}}$, where the degree of every node is $k_{0}$. By substituting $\langle k \rangle = k_{0}$ and $\langle k^{2} \rangle = k_{0}^{2}$ into Eq. (12), we arrive at the threshold for regular networks as:

13 $$\begin{aligned} \bar{\lambda }_{c}^{\text{SIRS}}(k_0 ) = \frac{1}{k_0 - ( \frac{h+2r}{h+r} ) } . \end{aligned}$$

Equations (12) and (13) accurately yield the known thresholds of the SI, SIR, and SIS models on quenched networks, as discussed below.

The SIRS model with $r=0$ corresponds to the SI model, where an endemic steady state of a positive *I* is possible for any $\lambda >0$, resulting in $\lambda _c^{\text{SI}} =0$. When $r=0$, $\lambda _{c}^{\text{SIRS}}$ is also zero, as expected. When *h* approaches infinity, the *R* state immediately transitions to *S*, effectively converting the SIRS model into the SIS model, whose threshold is given by $\bar{\lambda }^{\text{SIS}}_{c} = [\langle k \rangle /(\langle k^{2} \rangle - \langle k \rangle )]$. As *h* tends towards infinity, $\bar{\lambda }_{c}^{\text{SIRS}}$ approaches $\bar{\lambda }_{c}^{\text{SIS}}$. At $h=0$, the SIRS model simplifies to the SIR model, and the system ultimately stabilizes in a steady state where $R=N-S$, with $S\le S(t=0)$. In contrast to the absorbing phase transition seen in the SIRS model, the SIR model exhibits a threshold phenomenon briefly explained as follows^5^.

In a simple mean-field analysis, the rate equation for *I*(*t*) is described by $dI/dt = (\lambda S/N - r)I$. When the initial ratio *S*(0)/*N* is less than $\bar{\lambda }^{-1}$, the value of *I*(*t*) diminishes without increasing due to the decreasing susceptible population $S(t)<S(0)$. However, when $S(0)/N > \bar{\lambda }^{-1}$, *I*(*t*) initially increases to a maximum and then decays exponentially to zero. The inverse of $\bar{\lambda }$ is known as the basic reproduction number $R_0$. For the invasion of a disease into a population with $S(0)/N=1$, the disease will spread if the condition $R_0 > 1$ is met; otherwise, the disease will die out exponentially. Since the stationary number of recovered individuals *R* is given by $\int _{0}^{\infty } I(t)dt$, the SIR model displays a continuous transition from a state where $R=0$ to a state where $R>0$ at a finite value of $\bar{\lambda }_{c}^{\text{SIR}}$^4,31^. On quenched networks, the threshold $\bar{\lambda }_{c}^{\text{SIR}}$ is expressed as $[\langle k \rangle /(\langle k^{2} \rangle - 2 \langle k \rangle )]$, which agrees with $\bar{\lambda }_{c}^{\text{SIRS}}|_{h\rightarrow 0}$.

As a result, the threshold $\bar{\lambda }_{c}^{\text{SIRS}}$ is correctly reduced to the thresholds of the three well-known models: the SI, SIR, and SIS models, by considering the limits $r\rightarrow 0$, $h\rightarrow 0$, and $h\rightarrow \infty$, respectively.

In simulations, it is often convenient to work with rates normalized by their sum. In the subsequent discussion, $\lambda$, *r*, and *h* denote scaled rates and satisfy the conservation relation $\lambda + h + r = 1$. For simplicity, we will refer to $\lambda _{c}^{\text{SIRS}}$ as $\lambda _{c}$. By utilizing the relationship $\lambda + h + r = 1$, we can express $\lambda _{c}$ as a function of either *h* or *r* by eliminating *r* or *h* in Eq. (12)^36^. The resulting equations are as follows:

14 $$\begin{aligned} \lambda _c (h)= & {} \frac{\bar{\lambda }_{c}^{\text{SIS}}}{2\bar{\lambda }_{c}^{\text{HMF}}}\bigg (1-\sqrt{1-4(1-h) \frac{(\bar{\lambda }_{c}^{\text{HMF}})^{2} }{\bar{\lambda }_{c}^{\text{SIS}}}}\;\; \bigg ) , \end{aligned}$$

15 $$\begin{aligned} \lambda _c (r)= & {} \frac{1}{2}\bigg ( 1 - \sqrt{1-4r\bar{\lambda }_{c}^{\text{SIS}}} \bigg ) . \end{aligned}$$

Given that $\lambda _{c}(h)=\bar{\lambda }_{c}^{\text{SIS}}$ when $r=1-\lambda _c$ for $h=0$, $\lambda _{c}(h)$ monotonically decreases from $\bar{\lambda }_{c}^{\text{SIS}}$ to zero as *h* increases from 0 to 1. Regarding $\lambda _{c}(r)$, it is important to note that the upper limit of *r* is restricted to $r_\text{max} =1- \bar{\lambda }_{c}^{\text{SIS}}$, beyond which *h* becomes negative. As a result, $\lambda _{c}(r)$ increases from 0 to $\lambda _{c}(r_\text{max})$^36^.

**Figure 2:**
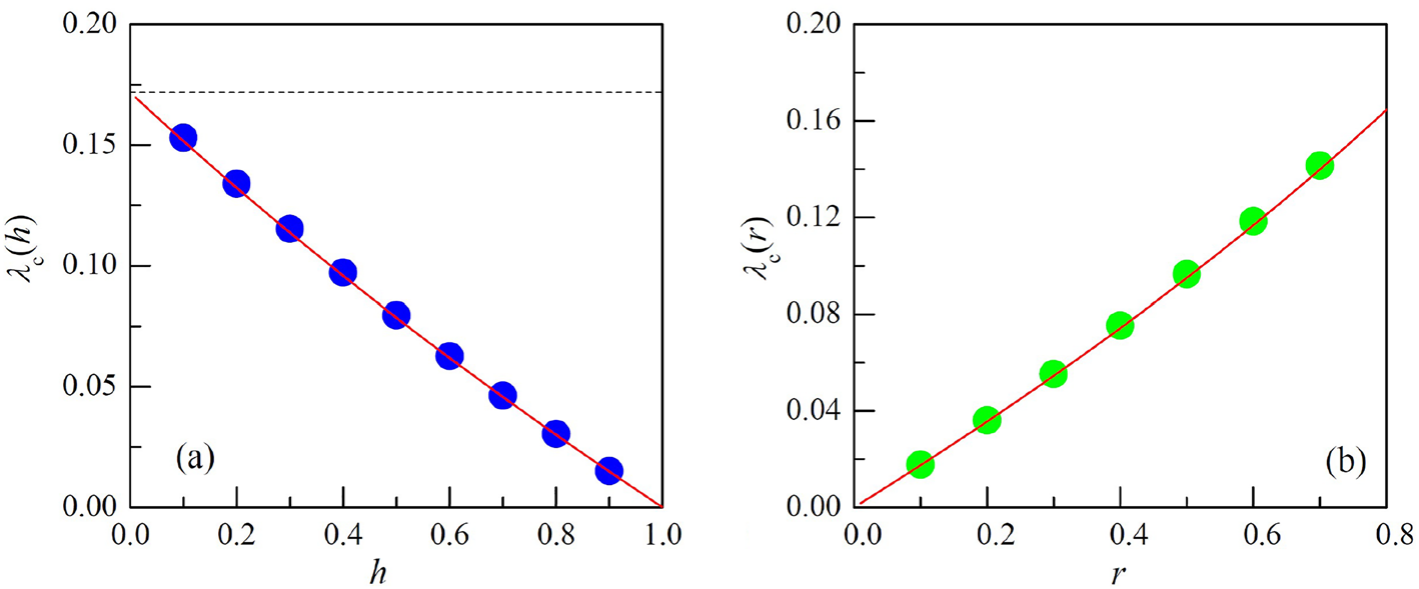
Phase Diagram of the SIRS Model on the scale-free network (SFN) with $\gamma =5$, $N=10^7$, $k_\text{min}=5$, and $k_\text{max}=100$: The SFN yields $\langle k \rangle = 6.094$ and $\langle k^2 \rangle = 41.547$, for which $\bar{\lambda }_{c}^{\text{SIS}}=0.1719$ and $\bar{\lambda }_{c}^{\text{HMF}}=0.14668$. (**a**) Plot of $\lambda _{c}(h)$ vs. *h*. The horizontal dashed line represents $\bar{\lambda }_{c}^{\text{SIR}}=\bar{\lambda }_{c}^{\text{SIS}}=0.1719$. (**b**) Plot of $\lambda _{c}(r)$ vs. *r*, where *r* is smaller than $r_\text{max}=1-\bar{\lambda }_{c}^{\text{SIS}} = 0.8281$ due to the constraint $h\ge 0$. In each panel, the circles represent the simulation-based estimates of $\lambda _{c}$, while the solid line corresponds to the theoretical $\lambda _{c}$ values derived from Eqs. (14) and (15). The discrepancies between the estimates and the theoretical values fall within a range of $0.9\%$ to $1.6\%$.

To validate the expressions for $\lambda _{c}(h)$ and $\lambda _{c}(r)$, we carried out Monte Carlo simulations of the SIRS model on quenched scale-free networks (SFNs) with $P(k)\sim k^{-\gamma }$ and draw the phase diagrams with the estimates of $\lambda _c$ obtained for various *h* and *r* as illustrated in Fig. 2 (Supplemental Material). Figure 2 shows the good agreement of the theoretical values and the estimates with high accuracy.

### CP model

The CP is another significant model that displays APTs on networks^4^ and in lattice structures^18–24^. In the CP, individuals undergo a cycle of infection dynamics as $S \overset{\lambda }{\rightarrow }\ I \overset{r}{\rightarrow }\ S$, analogous to the SIS model. However, a key distinction from the SIS model is that in the CP an infected individual randomly selects one of its neighbors and the infection occurs if the selected neighbor is susceptible, whereas in the SIS model an infected individual infects all susceptible neighbors^21–24^. Hence the transmission ways of the CP and SIS model are distinct. Previous attempts to calculate the threshold $\lambda ^{\text{CP}}_{c}$ for the CP were performed using the heterogeneous pair-approximation (HPA), which takes dynamic correlations and the random selection into account^35^. However, the HPA only provides a self-consistent equation for $\lambda ^{\text{CP}}_{c}$. Despite the similarity of the CP and SIS dynamics, an accurate expression for $\lambda ^{\text{CP}}_{c}$ on quenched networks has remained elusive.

**Figure 3:**
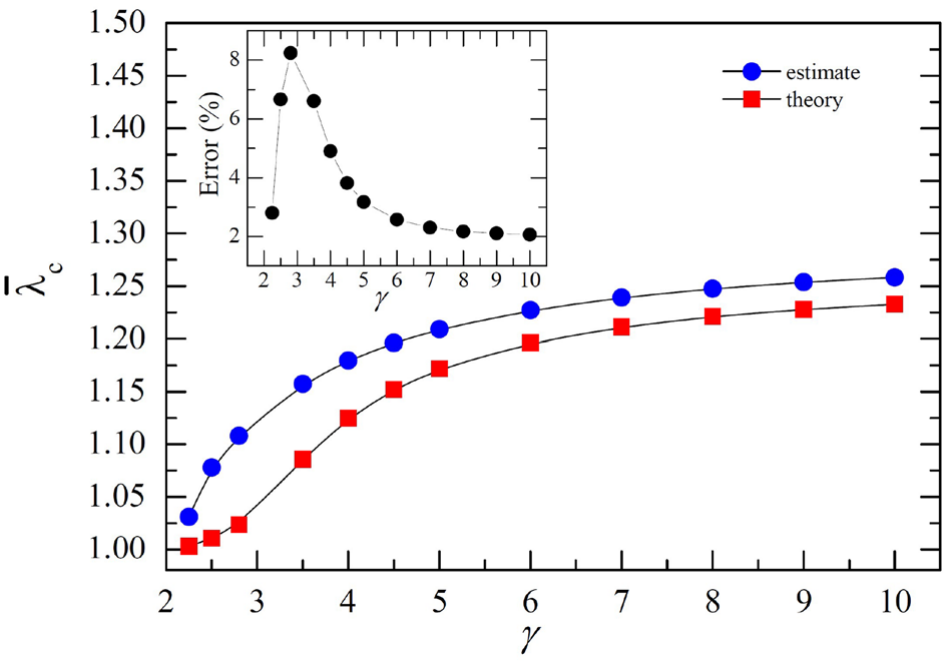
Phase diagram of the contact process (CP) on the scale-free network (SFN) with $N=10^7$ and $k_\text{min}=5$: Plots of $\bar{\lambda }_c$ vs. $\gamma$. Circles and squares are the estimates of $\bar{\lambda }_c$ resulting from simulations and the theoretical $\bar{\lambda }_c$ obtained from Eq. (17) for SFNs of various $\gamma$ ranging from 2.25, 2.5, 2.8, and 3.5 to 10. The inset displays the errors in the estimates compared to the theoretical values. A solid line has been drawn between the symbols to provide a visual guide.

The random selection make it impossible to write the HMF rate equation of $I_k$ analogous to Eq. (5) so that the protocol similar to the SIRS model cannot be applied to the CP. This problem can be resolved by rescaling the infection rate $\lambda$ with $\langle k \rangle /\langle k^2 \rangle$ and defining an effective infection rate $\Lambda _k = \lambda \langle k \rangle /\langle k^2 \rangle$ (Supplemental Material). Then we rewrite $dI_k /dt$ by approximating $p_k =p$ as

16 $$\begin{aligned} \frac{dI_k}{dt} = -r I_k + \Lambda _k kS_k p . \end{aligned}$$

Next, we obtain the protocol for the CP by dropping the variables related to $R_k$ in the protocol for the SIRS model. The BDB conditions for the CP are presented and confirmed by simulations in the Supplemental Material. By simply replacing $\Lambda _k$ with $\lambda$ in Eq. (16), we recover $dI_k /dt$ and the protocol for the SIS model^32^.

Subsequent procedures for deriving $\lambda ^{\text{CP}}_{c}$ are straightforward and yield the explicit expression for $\bar{\lambda }^{\text{CP}}_{c} (= \lambda ^{\text{CP}}_{c}/r)$^36^ as:

17 $$\begin{aligned} \bar{\lambda }^{\text{CP}}_{c} = \frac{\langle k^{2}\rangle }{\langle k^{2}\rangle - \langle k\rangle } . \end{aligned}$$

In the limit of $N\rightarrow \infty$, this expression yields $\bar{\lambda }^{\text{CP}}_{c}=1$ for $2<\gamma \le 3$, where $\langle k^2 \rangle$ diverges. In all other cases, $\bar{\lambda }^{\text{CP}}_{c}$ is greater than 1. For the regular network with degree $k_0$, we obtain $\bar{\lambda }^{\text{CP}}_{c} = k_0 /(k_0 -1 )$. The different expressions of $\bar{\lambda }^{\text{CP}}_{c}$ and $\bar{\lambda }^{\text{SIS}}_{c}$ are the consequence of the selection scheme of neighbors and show the importance of transmission ways in predicting the epidemic outbreak even for the same infection cycle $S \overset{\lambda }{\rightarrow }\ I \overset{r}{\rightarrow }\ S$.

We carried out simulations for the CP on quenched SFNs with $N=10^7$ and $k_\text{min}=5$, and estimated $\bar{\lambda }^{\text{CP}}_{c}$ for several $\gamma$ values. The resultant phase diagram is illustrated in Fig. 3 (Supplemental Material). As shown in the inset of Fig. 3, the error sharply increases near $\gamma =3$, which reflects that the network structure drastically changes due to diverging $\langle k^{2} \rangle$ at $\gamma =3$. For other values of $\gamma$, the errors are quite small, $2 \% \sim 4 \%$, so the simulation results convincingly support the expression of $\bar{\lambda }^{\text{CP}}_c$ of Eq. (17).

## Discussion

We have introduced a new approach involving BDB conditions and applied it to the ED approach in conjunction with the HMF theory to determine the epidemic thresholds for both the SIRS and CP models on quenched networks. The BDB conditions and the systematic method of rescaling infection rate make it possible to resolve the multiple stages of disease progression and the random selection of one neighbor, which have been the long-standing theoretical challenges in finding the accurate thresholds of the SIRS model and CP. We have derived the threshold of both models by combining the BDB conditions and the rescaling method with the approach developed by Cai *et el.*, and encapsulated the series of procedures for deriving thresholds in a general protocol. The predicted thresholds of both models according to the protocol were verified by simulations with high accuracy on quenched scale-free networks.

The SIRS and CP models are characterized by stationary cyclic behaviors. However, even for models that exhibit irreversible flows as their stationary behaviors, such as the SIR model, we can still derive the thresholds by introducing a virtual process that transforms such models into ones featuring cyclic stationary behaviors, similar to the SIRS model and taking the limit of the rate for the virtual process vanishing.

The utility of the protocol extends beyond these two specific models, as it is applicable to a wide range of epidemic models due to its general nature involving multiple stages of disease progression and various transmission ways. Given the successful derivation of epidemic thresholds for both the CP and SIRS models, our approach represented by the protocol is likely to yield accurate threshold expressions for epidemic models on diverse types of quenched networks, encompassing scenarios involving weighted, directed, and even directed-weighted networks.

Moreover, the application of our protocol to real-world epidemics would be intriguing. To implement our protocol, it is essential to first conduct model parameter estimation and analyze the network structure. Epidemic models, often characterized by non-linearities, are anticipated to have model parameters that vary over time and depend on numerous factors^37^. Consequently, the estimation of model parameters becomes challenging. This aspect could be considered in future works.

### Supplementary Information

Supplementary Information.

## Data Availability

All data generated and analyzed in this study are included in this article (and its Supplementary Information file).
