# Supplementary material for: General protocol for predicting outbreaks of infectious diseases in social networks
Source: Sci Rep. 2024 Mar 12;14:5973. doi: 10.1038/s41598-024-56340-7 (PMC10933380; doi:10.1038/s41598-024-56340-7)
Supplement: Supplementary file 1 — Supplementary Information. [file 41598_2024_56340_MOESM1_ESM.pdf]

Supplementary Information for "General protocol for predicting outbreaks of infectious diseases in social networks"

Sungchul Kwon and Jeong-Man Park
Department of Physics, The Catholic University of Korea, Bucheon 14662, Korea

In this Supplementary Information file, we provide additional information to complement the analytical details of our effective degree approach. We also present the algorithmic specifics of our Monte Carlo simulations, along with the derivation of the threshold and simulation outcomes for the contact process on quenched networks.

I. DETAILS FOR THE EFFECTIVE DEGREE APPROACH

A. Expressions for S_{kjn}, I_{kjn}, and R_{kjn}

We can express S_{kjn}, I_{kjn}, and R_{kjn} using binomial distributions involving p_k, q_k, and v_k for j infected neighbors, as well as w_k, x_k, and y_k for n recovered neighbors

$$S_{kjn} = S_k(1-p_k)^{k-j} p_k^j C_j^k (1-\hat{w}_k)^{k-j-n} \hat{w}_k^n C_n^{k-j} \quad (1)$$
$$I_{kjn} = I_k(1-q_k)^{k-j} q_k^j C_j^k (1-\hat{x}_k)^{k-j-n} \hat{x}_k^n C_n^{k-j} \quad (2)$$
$$R_{kjn} = R_k(1-v_k)^{k-j} v_k^j C_j^k (1-\hat{y}_k)^{k-j-n} \hat{y}_k^n C_n^{k-j} \quad (3)$$

where C_j^k represents a combination of k links taken j at a time without repetition and $\hat{w}_k = w_k/(1-p_k)$, $\hat{x}_k = x_k/(1-q_k)$, and $\hat{y}_k = y_k/(1-v_k)$ denote the conditional probabilities of a link being connected to a recovered neighbor given that the link is not connected to an infected neighbor. Subsequently, S_k, I_k, and R_k are calculated by summing S_{kjn}, I_{kjn}, and R_{kjn} across the range of j and n.

$$S_k = \sum_{j=0}^{k}\sum_{n=0}^{k-j} S_{kjn} = \sum_{j=0}^{k} S_{kj} \ , \ S_{kj} = \sum_{n=0}^{k-j} S_{kjn} \ ,$$
$$I_k = \sum_{j=0}^{k}\sum_{n=0}^{k-j} I_{kjn} = \sum_{j=0}^{k} I_{kj} \ , \ I_{kj} = \sum_{n=0}^{k-j} I_{kjn} \ ,$$
$$R_k = \sum_{j=0}^{k}\sum_{n=0}^{k-j} R_{kjn} = \sum_{j=0}^{k} R_{kj} \ , \ R_{kj} = \sum_{n=0}^{k-j} R_{kjn} \ .$$

We utilize abbreviated expressions for the summation notations such as $\sum_{kjn} = \sum_{k=k_{\min}}^{k_{\max}} \sum_{j=0}^{k} \sum_{n=0}^{k-j}$ and $\sum_{jn} = \sum_{j=0}^{k} \sum_{n=0}^{k-j}$.

B. Total infection, recovery, and loss-of-immunity rates

The total infection rate (σ), the total recovery rate (π), and the total loss-of-immunity rate (ϕ) in the population are defined as follows:

$$\sigma = \sum_{kjn} \lambda S_{kjn} j = \sum_k \lambda k p_k S_k, \quad (4)$$
$$\pi = \sum_{kjn} r I_{kjn} = \sum_k r I_k, \quad (5)$$
$$\phi = \sum_{kjn} h R_{kjn} = \sum_k h R_k. \quad (6)$$

In the steady state, all of these total rates become equal, $\sigma = \pi = \phi$, as indicated by Eq. (4) in the main text.

C. Bond-detailed-balance equations

From Table I in the main text, we derive six equations of the detailed balance conditions by summing the product of each contribution to $\Delta\Pi_{..}$ and the corresponding probability.

$$\langle \Delta\Pi_{SS}\rangle = -\sum_{kjn} \lambda S_{kjn} j\cdot(k-j-n) + \sum_{kjn} h R_{kjn}\cdot(k-j-n) = 0, \quad (7)$$

$$\langle \Delta\Pi_{II}\rangle = \sum_{kjn} \lambda S_{kjn} j\cdot j - \sum_{kjn} r I_{kjn}\cdot j = 0, \quad (8)$$

$$\langle \Delta\Pi_{RR}\rangle = \sum_{kjn} r I_{kjn}\cdot n - \sum_{kjn} h R_{kjn}\cdot n = 0, \quad (9)$$

$$\langle \Delta\Pi_{SI}\rangle = \textstyle\sum_{kjn} \lambda S_{kjn} j\cdot((k-j-n)-j) - \sum_{kjn} r I_{kjn}\cdot(k-j-n) + \sum_{kjn} h R_{kjn}\cdot j = 0, \quad (10)$$

$$\langle \Delta\Pi_{IR}\rangle = \textstyle\sum_{kjn} \lambda S_{kjn} j\cdot n + \sum_{kjn} r I_{kjn}\cdot(j-n) - \sum_{kjn} h R_{kjn}\cdot j = 0, \quad (11)$$

$$\langle \Delta\Pi_{RS}\rangle = -\textstyle\sum_{kjn} \lambda S_{kjn} j\cdot n + \sum_{kjn} r I_{kjn}\cdot(k-j-n) + \sum_{kjn} h R_{kjn}\cdot(n-(k-j-n)) = 0. \quad (12)$$

It is important to note that Cai *et al.* employed $\Delta\sigma = \Delta\pi = 0$ to derive Eq. (8), for the SIS model [12]. However, in the context of the SIRS model, Eq. (8) cannot be derived from $\Delta\sigma = \Delta\pi = 0$; it naturally emerges as one of the BDB conditions. Consequently, the BDB conditions play a crucial role in accounting for dynamic correlations.

D. Identities for the number of bonds with different types of nodes

The total number of infected neighbors of all susceptible nodes equals the total number of susceptible neighbors of all infected nodes, $\Pi_{SI} = \Pi_{IS}$, and the same applies for other bonds involving different types of nodes.

$$\Pi_{SI} = \Pi_{IS}: \quad \sum_{kjn} S_{kjn} j = \sum_{kjn} I_{kjn}(k-n-j) \quad (13)$$
$$\Pi_{SR} = \Pi_{RS}: \quad \sum_{kjn} S_{kjn} n = \sum_{kjn} R_{kjn}(k-n-j) \quad (14)$$
$$\Pi_{IR} = \Pi_{RI}: \quad \sum_{kjn} I_{kjn} n = \sum_{kjn} R_{kjn} j \quad . \quad (15)$$

E. Derivation of Eqs. (9) and (10) in the main text

To derive Eq. (9) in the main text, we begin by deriving the relation $\lambda \sum_{kjn} S_{kjn} j = r \sum_k I_k$.

$$\begin{aligned}
&\lambda \sum_{kjn} S_{kjn} j \\
&= \lambda \sum_{kjn} S_k (1-p_k)^{k-j} p_k^j {}_k C_j (1-\hat{w}_k)^{k-j-n} \hat{w}_k^n {}_{k-j} C_n \cdot j \\
&= \lambda \sum_{kj} S_k \bar{p}_k^{k-j} \left(p_k \frac{\partial}{\partial p_k} \right) p_k^j {}_k C_j \sum_{n=0}^{k-j} \bar{\hat{w}}_k^{k-j-n} \hat{w}_k^n {}_{k-j} C_n \\
&= \lambda \sum_k S_k \left(p_k \frac{\partial}{\partial p_k} \right) \bar{p}_k^{k-j} p_k^j {}_k C_j \left(\bar{\hat{w}}_k + \hat{w}_k \right)^{k-j} \\
&= \lambda \sum_k S_k \left(p_k \frac{\partial}{\partial p_k} \right) \left(\bar{p}_k \left(\bar{\hat{w}}_k + \hat{w}_k \right) + p_k \right)^k \\
&= \lambda \sum_k S_k p_k k \left(\bar{p}_k \left(\bar{\hat{w}}_k + \hat{w}_k \right) + p_k \right)^{k-1} \\
&= \lambda \sum_k k p_k S_k = r \sum_k I_k, \qquad (16)
\end{aligned}$$

where we used $\bar{p}_k = 1 - p_k$ and $\bar{\hat{w}}_k = 1 - \hat{w}_k$. Based on this, we can derive the following relation.

$$\begin{aligned}
&\lambda \sum_{kjn} S_{kjn} jk = \lambda \sum_k k \cdot \sum_{jn} S_{kjn} j = \lambda \sum_k k \cdot k p_k S_k \\
&= \lambda \sum_k k \cdot \frac{r}{\lambda} I_k = r \sum_{kjn} I_{kjn} k. \qquad (17)
\end{aligned}$$

By utilizing this relation and the identity $\Pi_{IR} = \Pi_{RI}$, Eq. (15) above, we arrive at Eq. (9) as presented in the main text.

We transform Eq. (9) in the main text into an equation involving only I_k, which allows us to determine the threshold. We express the second and third terms Eq. (9) in the main text with I_k by using the similar procedure as described for Eq. (16).

$$\begin{aligned}
&\lambda \sum_{kjn} S_{kjn} j^2 \\
&= \lambda \sum_k S_k \left(p_k \frac{\partial}{\partial p_k} \right)^2 \left(\bar{p}_k \left(\bar{\hat{w}}_k + \hat{w}_k \right) + p_k \right)^k \\
&= \lambda \sum_k S_k \left(p_k \frac{\partial}{\partial p_k} \right) \left[p_k k \left(\bar{p}_k \left(\bar{\hat{w}}_k + \hat{w}_k \right) + p_k \right)^{k-1} \right] \\
&= \lambda \sum_k S_k p_k k \left[\left(\bar{p}_k \left(\bar{\hat{w}}_k + \hat{w}_k \right) + p_k \right)^{k-1} \right. \\
&\quad \left. + p_k (k-1) \left(\bar{p}_k \left(\bar{\hat{w}}_k + \hat{w}_k \right) + p_k \right)^{k-2} \right] \\
&= \lambda \sum_k S_k k p_k \left[1 + p_k (k-1) \right] \\
&= r \sum_k I_k \left[1 + p_k (k-1) \right]. \qquad (18)
\end{aligned}$$

Similarly, we proceed with the calculation as follows:

$$\begin{aligned}
&\lambda \sum_{kjn} S_{kjn} jn \\
&= \lambda \sum_k S_k \left(p_k \frac{\partial}{\partial p_k} \right) \left(\hat{w}_k \frac{\partial}{\partial \hat{w}_k} \right) \left(\bar{p}_k \left(\bar{\hat{w}}_k + \hat{w}_k \right) + p_k \right)^k \\
&= \lambda \sum_k S_k \left(\hat{w}_k \frac{\partial}{\partial \hat{w}_k} \right) \left[p_k k \left(\bar{p}_k \left(\bar{\hat{w}}_k + \hat{w}_k \right) + p_k \right)^{k-1} \right] \\
&= \lambda \sum_k S_k k p_k \hat{w}_k (k-1) \left(\bar{p}_k \left(\bar{\hat{w}}_k + \hat{w}_k \right) + p_k \right)^{k-2} \bar{p}_k \\
&= \lambda \sum_k S_k k (k-1) p_k (1-p_k) \hat{w}_k \\
&= r \sum_k I_k (k-1)(1-p_k) \hat{w}_k = r \sum_k I_k (k-1) w_k, \qquad (19)
\end{aligned}$$

where we used $\hat{w}_k = w_k/(1-p_k)$.

For expressing the first term in terms of I_k, we need to use the identities $\Pi_{SI} = \Pi_{IS}$, Eq. (13). By utilizing Eqs. (8) and (13), the first term of Eq. (9) in the main text can be expressed as:

$$\sum_{kjn} I_{kjn} n = \sum_k k I_k - \frac{r}{\lambda} I - \frac{\lambda}{r} \sum_{kjn} S_{kjn} j^2 \quad . \qquad (20)$$

Substituting Eqs. (18), (19), and (20) into Eq. (9) in the main text, we can rewrite it as Eq. (10) in the main text:

$$\begin{aligned}
&(r+h) \left(\sum_k k I_k - \tfrac{r}{\lambda} \sum_k I_k \right) \\
&-(2r+h) \sum_k \left(1 + p_k (k-1) \right) I_k - r \sum_k w_k (k-1) I_k = 0 \quad .
\end{aligned}$$

F. Derivation of epidemic threshold

We define the left-hand side of Eq. (10) in the main text as $f(p)$ and represent $f(p)$ compactly as $f(p) =$

$L\sum_k I_k + H\sum_k kI_k$ by introducing the definitions of H and L:

$$\begin{aligned} H &= (h+r) - (h+2r)p - rw(p), \\ L &= -\frac{r}{\lambda}(h+r) - (h+2r)(1-p) + rw(p). \end{aligned} \quad (21)$$

By differentiating $f(p)$ and using the relations

$$\sum_k \frac{dI_k}{dp}\bigg|_{p=0} = \frac{\lambda N\langle k\rangle}{r}, \quad \sum_k k\frac{dI_k}{dp}\bigg|_{p=0} = \frac{\lambda N\langle k^2\rangle}{r}, \quad (22)$$

we derive the equation for λ_c:

$$\bigg(r(h+r) + \lambda_c(h+2r)\bigg)\langle k\rangle - \lambda_c(h+r)\langle k^2\rangle = 0 \ , \quad (23)$$

leading to the epidemic threshold $\bar{\lambda}_c = \lambda_c/r$ of the SIRS model as

$$\bar{\lambda}_c^{\mathrm{SIRS}} = \frac{\langle k\rangle}{\langle k^2\rangle - (\frac{h+2r}{h+r})\langle k\rangle} \ .$$

II. DETAILS FOR SIMULATIONS

A. Scaled rates for simulations

In the main text, we derived the expression for $\bar{\lambda}_c^{\mathrm{SIRS}}$ as

$$\bar{\lambda}_c^{\mathrm{SIRS}} = \frac{\langle k\rangle}{\langle k^2\rangle - (\frac{h+2r}{h+r})\langle k\rangle} \ . \quad (24)$$

For simulation purposes, it is more convenient to express $\bar{\lambda}_c^{\mathrm{SIRS}}$ in terms of scaled rates, which satisfy the conservation relation $\lambda + h + r = 1$. In the following subsections, we will use λ, r, and h as the probabilities for the infection, recovery, and loss-of-immunity processes, respectively.

First, we can simplify the equation by eliminating r from Eq. (24) using $r = 1 - \lambda_c - h$, which yields the following equation:

$$(\langle k^2\rangle - \langle k\rangle)\lambda_c^2 - \langle k^2\rangle\lambda_c + (1-h)\langle k\rangle = 0 \ . \quad (25)$$

Now, we define the coefficients as follows:

$$x = \langle k^2\rangle - \langle k\rangle, \ y = \langle k^2\rangle, \ z = \langle k\rangle(1-h) \ . \quad (26)$$

Given that $\langle k^2\rangle \gg \langle k\rangle$ and $h \leq 1$, all three coefficients are positive. Consequently, we obtain a second-order equation in terms of λ_c:

$$x\lambda_c^2 - y\lambda_c + z = 0 \ . \quad (27)$$

The solutions for λ_c are then given by:

$$\lambda_c(h) = \frac{y}{2x}\left(1 \pm \sqrt{1 - \frac{4xz}{y^2}}\right) \ . \quad (28)$$

By using the relation $\bar{\lambda}_c^{\mathrm{HMF}} = \langle k\rangle/\langle k^2\rangle$, we can rewrite $4xz/y^2$ as $4(1-h)\bar{\lambda}_c^{\mathrm{HMF}}(1-\bar{\lambda}_c^{\mathrm{HMF}})$. This quadratic function of $\bar{\lambda}_c^{\mathrm{HMF}}$ has its maximum value of $(1-h)$ at $\bar{\lambda}_c^{\mathrm{HMF}} = 1/2$. Since $4xz/y^2 \in [0,1]$ for $0 \leq h \leq 1$, the square root will fall within the range $[0,1]$, ensuring that $\lambda_c(h)$ remains real. To satisfy the condition $\lambda_c(h) = \bar{\lambda}_c^{\mathrm{SIR}}$ for $h = 0$, we should consider the negative solution. Thus, we can express it as:

$$\lambda_c(h) = \frac{\bar{\lambda}_c^{\mathrm{SIS}}}{2\bar{\lambda}_c^{\mathrm{HMF}}}\left(1 - \sqrt{1 - 4(1-h)\frac{(\bar{\lambda}_c^{\mathrm{HMF}})^2}{\bar{\lambda}_c^{\mathrm{SIS}}}}\ \right) \ , \quad (29)$$

where $\bar{\lambda}_c^{\mathrm{SIS}} = \langle k^2\rangle/(\langle k^2\rangle - \langle k\rangle)$.

For $h = 0$, the SIRS model is reduced to the SIR model. However, if we plug in $h = 0$ into Eq. (24), we obtain $\lambda_c = \bar{\lambda}_c^{\mathrm{SIS}}$, which can also be derived by substituting $r = 1 - \lambda_c$ into the expression $\bar{\lambda}_c^{\mathrm{SIR}} = \langle k^2\rangle/(\langle k^2\rangle - 2\langle k\rangle)$. This leads us to $\lambda_c(h=0) = \bar{\lambda}_c^{\mathrm{SIR}}|_{r=1-\lambda_c} = \bar{\lambda}_c^{\mathrm{SIS}}$ due to the conservation $\lambda_c + r = 1$. For $h = 1$, the conservation implies $\lambda_c = 0$ for the SI model, since $r = 0$. Consequently, $\lambda_c(h)$ monotonically decreases from $\bar{\lambda}_c^{\mathrm{SIR}}$ to the SI threshold $\bar{\lambda}_c^{\mathrm{SI}}$ as h increases from 0 to 1.

Next, we eliminate h from Eq. (24), resulting in the following equation for λ_c:

$$\lambda_c^2 - \lambda_c + r\bar{\lambda}_c^{\mathrm{SIS}} = 0 \ . \quad (30)$$

Solving Eq. (30), we obtain $\lambda_c(r)$ as follows:

$$\lambda_c(r) = \frac{1}{2}\left(1 \pm \sqrt{1 - 4r\bar{\lambda}_c^{\mathrm{SIS}}}\right) \ . \quad (31)$$

However, the positive solution violates the condition $\lambda_c = \bar{\lambda}_c^{\mathrm{SI}} = 0$ for $r = 0$. By considering the negative solution, we can express $\lambda_c(r)$ as:

$$\lambda_c(r) = \frac{1}{2}\left(1 - \sqrt{1 - 4r\bar{\lambda}_c^{\mathrm{SIS}}}\right) \ . \quad (32)$$

For the square root to yield a real value, r should satisfy the following condition:

$$r \leq \frac{1}{4\bar{\lambda}_c^{\mathrm{SIS}}} \ . \quad (33)$$

Additionally, from $h = 1 - \lambda_c - r \geq 0$, we require:

$$h = \frac{1}{2} - r + \frac{1}{2}\sqrt{1 - 4r\bar{\lambda}_c^{\mathrm{SIS}}} \geq 0 \ . \quad (34)$$

We can rephrase the inequality of Eq. (34) in terms of r as:

$$r \ \leq \ 1 - \bar{\lambda}_c^{\mathrm{SIS}} \ . \quad (35)$$

Consequently, r should satisfy one of the two conditions, either Eq. (33) or Eq. (35), depending on the magnitude of $\bar{\lambda}_c^{\mathrm{SIS}}$. However, for $\bar{\lambda}_c^{\mathrm{SIS}} \leq 1$, it can be demonstrated that $(1 - \bar{\lambda}_c^{\mathrm{SIS}}) \leq (4\bar{\lambda}_c^{\mathrm{SIS}})^{-1}$ by comparing Eqs. (33) and (35), where equality holds at $\bar{\lambda}_c^{\mathrm{SIS}} = 1/2$. This means

that the inequality of Eq. (35) must be satisfied by r, restricting the maximum r ($r_{\max}$) as $r_{\max} = 1 - \bar{\lambda}_c^{\mathrm{SIS}}$.

It is important to note that $\lambda_c = \lambda_c^{\mathrm{SI}} = 0$ when $r = 0$, as expected. As r increases, $\lambda_c(r)$ monotonically increases from 0 to the maximum value $r_{\max}$, above which h becomes negative.

B. The uncorrelated configuration (UC) algorithm for the generation of networks

In the quenched scale-free network (SFN) characterized by a degree distribution $P(k) \sim k^{-\gamma}$, the network is designed with predetermined minimum and maximum degrees, represented as $k_{\min}$ and $k_{\max}$, respectively. The procedure for constructing these networks adheres to the algorithm outlined in Refs. [1, 2].

The number of nodes with a degree of k, denoted as N_k, is determined through the following deterministic calculation: $N_k = \mathrm{int}[\sum_{k' \geq k}^{k_{\max}} NP(k')] - \mathrm{int}[\sum_{k' \geq (k+1)}^{k_{\max}} NP(k')]$. This is done while ensuring that the total number of nodes, denoted as N, adheres to the equation $N = \sum_{k=k_{\min}}^{k_{\max}} N_k$. Here, the notation $\mathrm{int}[x]$ signifies the integer part of the real number x. To allocate a degree of k to N_k nodes, a random selection is made from the pool of nodes that have not yet been assigned degrees. Subsequently, the selected N_k nodes are assigned the degree k. By repeating these steps for all integer values of k within the range $[k_{\min}, k_{\max}]$, each node is allocated a specific degree. This process generates the degree sequence $\mathbf{K}_N = k_1, ..., k_N$ for all N nodes.

Finally, to create a quenched network, a procedure is employed where two nodes are chosen at random. The crucial requirement for this selection is that the two chosen nodes are not already linked and that their current degrees do not surpass their designated degrees as per the degree sequence $\mathbf{K}_N$. If these conditions are met, the two nodes are linked together. However, if the conditions are not satisfied, an alternative pair of nodes that adheres to the conditions is randomly chosen. This process of linking nodes continues until all nodes are connected to one another according to the predefined degree sequence $\mathbf{K}_N$.

Since the algorithm used is deterministic, all the generated quenched networks follow the same degree sequence $\mathbf{K}_N$. The sole variation among these networks lies in the connections formed between nodes. The process of randomly rewiring the connections of N nodes only results in the rearrangement of linked nodes, and it doesn't impact the distribution $P(k)$. As a result, networks characterized by the same degree sequence $\mathbf{K}_N$ yield identical network-level averages for any observable X, expressed as $\langle X \rangle = \sum_k XP(k)$. Hence, during our simulations, we utilize a single network and compute ensemble averages across multiple samples, commencing from distinct initial distributions yet sharing the same initial density of I and S.

C. The SIRS on a quenched SFN of $\gamma > 4$

To validate the expressions for $\lambda_c(h)$ and $\lambda_c(r)$ as given by Eqs. (29) and (32), we perform simulations of the SIRS model on quenched SFNs.

We construct a scale-free network (SFN) with a power-law degree distribution characterized by $\gamma = 5$, $N = 10^7$, $k_{\min} = 5$, and $k_{\max} = 100$. The uncorrelated configuration (UC) algorithm detailed earlier is used to generate this network configuration. The resulting network has an average degree $\langle k \rangle = 6.0942$ and an average squared-degree $\langle k^2 \rangle = 41.547$. For this particular network configuration, we have the following values for $\bar{\lambda}_c^{\mathrm{HMF}}$ and $\bar{\lambda}_c^{\mathrm{SIS}}$:

$$\bar{\lambda}_c^{\mathrm{HMF}} = 0.1467 \ , \ \ \bar{\lambda}_c^{\mathrm{SIS}} = 0.172 \ . \tag{36}$$

With these parameters in place, we proceed with simulations to analyze and verify the expressions for $\lambda_c(h)$ and $\lambda_c(r)$.

For this quenched SFN, simulations are conducted using a sequential updating scheme. The process involves creating a list of infected and recovered nodes, followed by the random selection of a node from the list. If the chosen node is infected, it transitions to the recovered state with a probability r ($I \to R$). On the other hand, if the chosen node is susceptible, all susceptible nodes linked to the infected node may become infected with a probability λ ($S \to I$). If a recovered node is selected, it transitions to the susceptible state with a probability h ($R \to S$). The list is updated in response to changes brought about by the $S \to I$ and $R \to S$ processes. Importantly, the $I \to R$ process does not alter the list.

After updating the list, the Monte Carlo time t is incremented by $dt = 1/M(t)$, where $M(t)$ represents the total number of infected and recovered nodes, i.e., the size of the list, before the update. The initial conditions are established by randomly infecting $I(0)$ nodes in the entirely susceptible population of size N, which results in $S(0) = N - I(0)$ and $R(0) = 0$. For a specific initial condition defined by a given $I(0)$ value, we run simulations until a predefined time $t_{\max}$ and calculate the averages of the quantities $S(t)$, $I(t)$, and $R(t)$.

Initially, we outline the process used to estimate λ_c for $h = 0.5$ and $r = 0.5$. Subsequently, we compare these estimates with the theoretical values of $\lambda_c(h)$ and $\lambda_c(r)$, which are provided by Eqs.(29) and (32), corresponding to Eqs.(14) and (15) in the main text. Finally, we create two phase diagrams, namely $\lambda_c(h)$ versus h and $\lambda_c(r)$ versus r, based on the obtained estimates for h in the range of $[0.1, 0.9]$ and r in the range of $[0.1, 0.7]$.

(1) $\lambda_c(h)$ for $h = 0.5$

To investigate the off-critical scaling behavior, we conduct simulations up to $t_{\max} = 10^4$ using random initial conditions of $S(0) = I(0) = 0.5$. The quantity $I(\Delta, t)$ is averaged over approximately 50 samples, and simulations are carried out for various values of λ ranging from 0.081 to 0.089. To obtain the steady-state

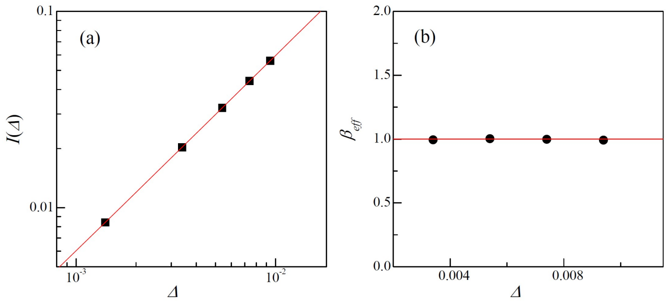

FIG. 1: $I(\Delta)$ for $h = 0.5$: Plots of $I(\Delta)$ and $\beta_{\rm eff}$ vs. $\Delta = \lambda - \lambda_c$ with $\lambda_c = 0.0796$. (a) Each symbol corresponds to the value of $I(\Delta)$ for different λ values: 0.081, 0.083, 0.085, 0.087, and 0.089. The red straight line represents the fitting line with a slope of unity, indicating $\beta = 1$. (b) Throughout the plot, $\beta_{\rm eff}$ remains consistently near the value of 1.

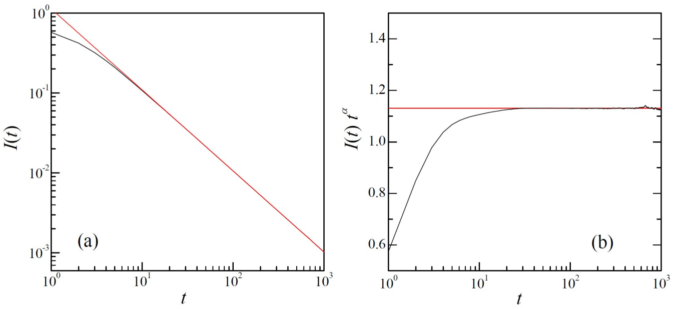

FIG. 2: The critical decay for $h = 0.5$: (a) Plot of the critical decay of $I(t)$ at $\lambda = 0.0796$. The red straight line corresponds to the fitting line with a slope of unity. (b) The scaling plot of $f(t, \alpha) = I(t)t^{\alpha}$ with $\alpha = 1.014$. The horizontal line indicates the average value of $f(t, \alpha)$ in the scaling region.

value $I(\Delta)$, we perform a time average within an interval of $[\tau, t_{\rm max}]$, where τ denotes the time after which $I(\Delta, t)$ stabilizes to the steady state.

We investigate the power-law decay of $I(\Delta)$ with respect to Δ as Δ^{β} for $\Delta \to 0$. To determine λ_c, we examine the linearity of $I(\Delta)$ by varying a tentative value λ_c^* in a double logarithmic plot of I against Δ (where $\Delta = \lambda - \lambda_c^*$). If we adjust λ_c^* to be either larger or smaller than the true λ_c, the plot of $I(\Delta)$ displays nonlinearity as Δ approaches zero. As a result, we estimate λ_c by identifying the λ_c^* value that results in a linear $I(\Delta)$ plot and calculate the slope of the least-square fitting line to determine the exponent β.

In Fig. 1(a), the double logarithmic plot of I against Δ at $\lambda_c^* = 0.0796$ is depicted, revealing the clear linear decay of I as Δ approaches zero. This observation leads us to estimate λ_c as 0.0796 for $h = 0.5$. The theoretical value of $\lambda_c(h)$, denoted as $\lambda_c^{\rm th}(h)$, for $h = 0.5$ is $\lambda_c^{\rm th}(h = 0.5) = 0.07861$. While our estimate is slightly higher than $\lambda_c^{\rm th}(h)$, the error remains minimal at only 1.253%.

By applying the least-square fitting to the data in Fig. 1(a), we calculate the slope $\beta = 0.9970(9)$. The value in the parentheses signifies the error associated with the last digit of the fitting result. To ensure the stability of the slope estimation, we compute the effective slope $\beta_{\rm eff}$ between consecutive data points, and the results are plotted in Fig. 1(b). As evident from the figure, $\beta_{\rm eff}(\Delta)$ saturates and remains consistently close to 1, thereby confirming the precision of the estimated λ_c.

To further verify the accuracy of $\lambda_c = 0.0796$, we approach the estimation through an alternative method. We examine the critical decay of $I(t) \sim t^{-\alpha}$ at $\lambda_c = 0.0796$. In this procedure, simulations are conducted up to $t_{\rm max} = 10^3$, commencing from random initial conditions with $S(0) = 0.2$ and $I(0) = 0.8$, and the average $I(t)$ is computed over 1000 samples.

Fig. 2(a) presents a double logarithmic plot of $I(t)$ against t. As clearly depicted, $I(t)$ exhibits a decay consistent with $t^{-\alpha}$ behavior following an initial transient time. To ascertain the decay exponent α, we analyze the scaling plot of $f(t, \alpha) = I(t)t^{\alpha}$, where α is varied. An accurate choice of α results in a constant value of $f(t, \alpha)$ within a scaling region governed by $I(t) \sim t^{-\alpha}$. As shown in Fig. 2(b), we observe that $f(t, \alpha)$ achieves a flat plateau for $\alpha = 1.014$, which reinforces the precision of the estimated $\lambda_c = 0.0796$. Consequently, we confidently determine the estimates for $h = 0.5$ as $\lambda_c = 0.0796$ and $\alpha = \beta = 1$ for $\gamma = 5$.

(2) λ_c for $r = 0.5$

Similarly to the $h = 0.5$ case, we adopt a similar approach to estimate λ_c for $r = 0.5$. This involves examining the off-critical scaling behavior of $I(\Delta)$ and subsequently confirming the accuracy of the estimated λ_c through the critical decay of $I(t)$.

The simulations are initiated with various λ values ranging from 0.0975 to 0.101. The simulations begin with random initial conditions of $S(0) = I(0) = 0.5$ and continue up to $t_{\rm max} = 3 \times 10^4$, the precise value depending on λ. The quantity $I(\Delta)$ is averaged over a span of up to 200 samples.

By fine-tuning λ_c^* in the double logarithmic plot of $I(\Delta)$, the optimal linearity of $I(\Delta)$ is achieved at $\lambda_c^* = 0.09646$. Fig. 3 depicts the double logarithmic plot of $I(\Delta)$ and $\beta_{\rm eff}(\Delta)$ for this value of λ_c^*. By analyzing the slope of the least-square fitting line in Fig. 3(a), we estimate $\lambda_c = 0.09646$ and $\beta = 1.00(1)$. Furthermore, Fig. 3(b) demonstrates that $\beta_{\rm eff}(\Delta)$ remains consistently close to 1, providing additional confirmation of the precision of the estimated λ_c.

To confirm the power-law decay at the estimated λ_c, simulations were conducted at $\lambda_c = 0.09646$ with a runtime of up to $t_{\rm max} = 500$. These simulations began with random initial conditions of $S(0) = I(0) = 0.5$, and $I(t)$ was averaged over 1000 samples. As depicted in Fig. 4(a), $I(t)$ exhibited a power-law decay pattern after a brief transition period. Utilizing the scaling plot showcased in Fig. 4(b), an estimate of $\alpha = 0.992$ was derived.

Based on the findings from the analyses of off-critical and critical scaling behaviors of I, it can be concluded

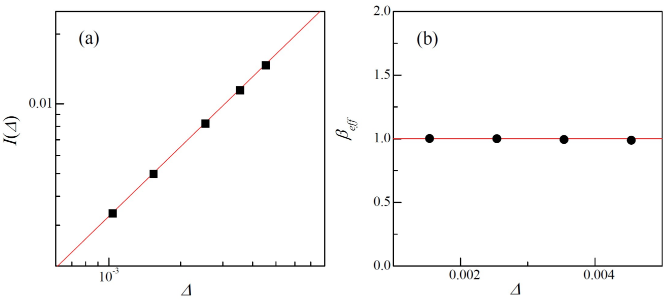

FIG. 3: $I(\Delta)$ for $r = 0.5$: Plots of $I(\Delta)$ and $\beta_{\rm eff}$ vs. $\Delta = \lambda - \lambda_c$ with $\lambda_c = 0.09646$. (a) Each symbol corresponds to $I(\Delta)$ for $\lambda = 0.0975$, 0.098, 0.099, 0.1, and 0.101. The straight red line represents the fitting line with a unit slope, indicating $\beta = 1$. (b) The values of $\beta_{\rm eff}$ remain consistently close to 1.

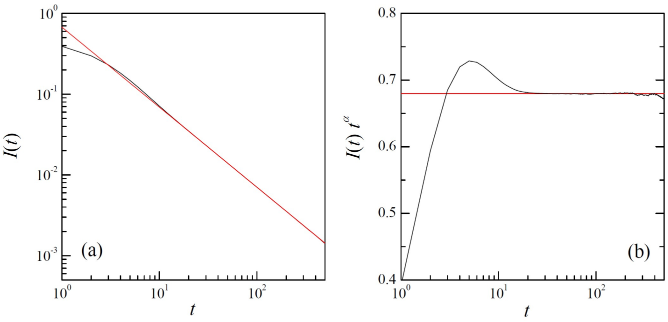

FIG. 4: The critical decay for $r = 0.5$: (a) Plot of the critical decay of $I(t)$ at $\lambda = 0.09646$. The red line is a fitting line with a unit slope. (b) The scaling plot of $f(t, \alpha) = I(t)t^{\alpha}$ with $\alpha = 0.992$. The horizontal line indicates the average value of $f(t, \alpha)$ in the scaling region.

that the estimates for $r = 0.5$ are $\lambda_c = 0.09646$ and $\alpha = \beta = 1$ for a network with $\gamma = 5$. The theoretical value of $\lambda_c(r)$ ($\lambda_c^{\rm th}(r)$) for $r = 0.5$ is $\lambda_c^{\rm th}(r = 0.5) = 0.09497$. Thus, the presented estimate is only 1.583% higher than the value of $\lambda_c^{\rm th}(r)$.

(3) The phase diagrams

In a manner analogous to the estimation processes for $h = 0.5$ and $r = 0.5$, we perform estimations for $\lambda_c(h)$ by incrementally increasing h from 0.1 to 0.9 in increments of 0.1. Additionally, we estimate $\lambda_c(r)$ by increasing r from 0.1 to 0.7 in increments of 0.1. It is important to note that r is constrained by the maximum value of r ($r_{\rm max}$) given by $r_{\rm max} = 1 - \bar{\lambda}_c^{\rm SIS} = 0.8281$, as determined by $\bar{\lambda}_c^{\rm SIS}$ of Eq. (36). Values of r exceeding this limit result in $h \leq 0$.

We have constructed the phase diagrams using the estimates of $\lambda_c(h)$ and $\lambda_c(r)$, as shown in the plot of $\lambda_c(h)$ versus h (Fig. 5(a)) and the plot of $\lambda_c(r)$ versus r (Fig. 5(b)). In both diagrams, one region where $\lambda > \lambda_c$ corresponds to the active/endemic phase, while the other region where $\lambda < \lambda_c$ corresponds to the absorbing/disease-free phase. Notably, all the estimates are in close proximity to the theoretical lines obtained through Eqs. (29) and (32), which correspond to Eqs. (14) and (15) in the main text. The errors in the estimates from the theoretical values are minimal, measuring less than 1.3% for $\lambda(h)$ and less than 1.584% for $\lambda(r)$, respectively. Consequently, all the estimated λ_c values demonstrate excellent agreement with the theoretical predictions.

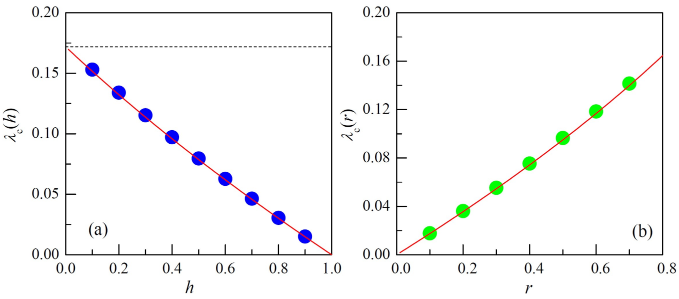

FIG. 5: Phase diagram of the SIRS model on the quenched SFN with $\gamma = 5$, $N = 10^7$, $k_{\rm min} = 5$, and $k_{\rm max} = 100$: Plots of $\lambda_c(h)$ vs. h (a) and $\lambda_c(r)$ vs. r (b). In (a), the horizontal dashed line indicate $\bar{\lambda}_c^{\rm SIR} = \bar{\lambda}_c^{\rm SIS} = 0.1719$. In (b), r is less than $r_{\rm max} = 1 - \bar{\lambda}_c^{\rm SIS} = 0.8281$, above which $h \leq 0$. In each panel, the circles represent the estimates of λ_c obtained from simulations, while the solid lines denote the theoretical λ_c values derived from Eqs. (29) and (32), using the substitutions of $\bar{\lambda}_c^{\rm HMF}$ and $\bar{\lambda}_c^{\rm SIS}$ from Eq. (36). The discrepancies between the estimates and theoretical values fall within the range of approximately 0.9% to 1.6%.

For the SIS model, both the HMF theory and the mean-field Langevin equation predict $\beta^{\rm HMF} = 1$ when $\gamma > 4$, and $\beta^{\rm HMF} = 1/(\gamma - 3)$ for $3 < \gamma < 4$ and $\alpha = \beta^{\rm HMF}$ [7–9]. Applying the HMF theory to the SIRS model yielded the same results as those observed in the SIS model, indicating that the state R does not alter the mean-field exponents. Remarkably, our simulation results of $\alpha = \beta = 1$ for $\gamma = 5$ align well with the predictions of the HMF theory for $\gamma > 4$.

D. The SIRS on a quenched SFN of $3 < \gamma < 4$

We generate a scale-free network with $\gamma = 3.5$, comprising $N = 10^8$ nodes. The minimum and maximum degree values for this network are set as $k_{\rm min} = 5$ and $k_{\rm max} = 5724$, respectively. The network construction procedure results in average degree values $\langle k \rangle = 7.5751$ and $\langle k^2 \rangle = 100.1724$. Based on these values, we calculate the corresponding $\bar{\lambda}_c^{\rm HMF}$ and $\bar{\lambda}_c^{\rm SIS}$ as provided in Eq. (37), namely:

$$\bar{\lambda}_c^{\rm HMF} = 0.07562 \ , \ \ \bar{\lambda}_c^{\rm SIS} = 0.08181 \ . \qquad (37)$$

For $h = 0.5$ and 0.9, we follow a similar process to estimate $\lambda_c(h)$ as previously done for $\gamma = 5$.

For $h = 0.5$, we increase λ by 5×10^{-4} within the range of 0.0405 to 0.0425. Subsequently, we compute

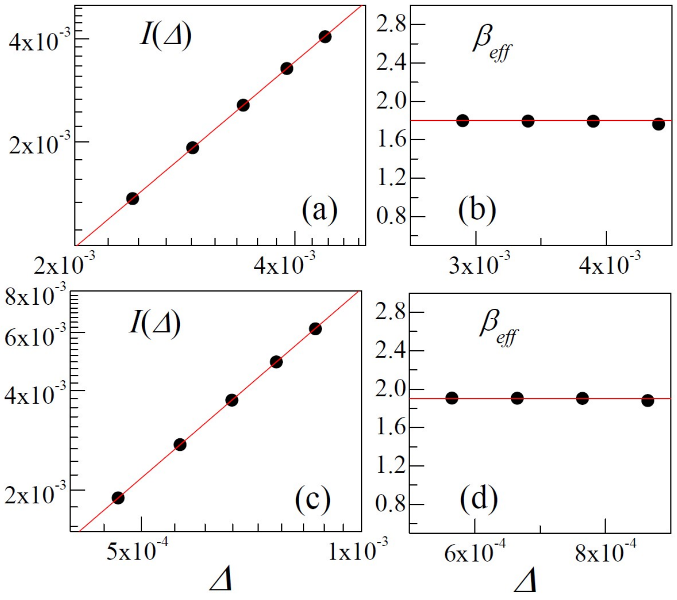

FIG. 6: $\gamma = 3.5$: Plots of $I(\Delta)$ and $\beta_{\rm eff}$ vs. Δ with $\lambda_c = 0.0381$ for $h = 0.5$ (a) and (b), and with $\lambda_c = 0.007235$ for $h = 0.9$ (c) and (d) respectively. In panel (a) and (c), the straight lines are the least-square fitting line with slope 1.796 for $h = 0.5$ (a) and 1.9051 for $h = 0.9$ (c). In panel (b) and (d), the horizontal lines indicate $\beta_{\rm eff} = 1.8$ (b) and 1.9 (d).

$I(\Delta, t)$ up to $t_{\rm max} = 10^4$ and then average I over up to 160 samples. The simulations are initiated with random initial conditions, where both $I(0)$ and $S(0)$ are set to 0.5.

After adjusting λ_c^*, we determine the optimal linearity of $I(\Delta)$ at $\lambda_c^* = 0.0381$. Fig. 6(a) and 6(b) illustrate the double logarithmic plots of $I(\Delta)$ and $\beta_{\rm eff}(\Delta)$ corresponding to $\lambda_c^* = 0.0381$. In particular, $\beta_{\rm eff}(\Delta)$ remains consistently stable around 1.8, allowing us to estimate $\lambda_c = 0.0381$ and $\beta = 1.796(1)$ using the slope of the least-square fitting line in Fig. 6(a).

For $\lambda_c = 0.0381$, we perform simulations using 180 samples, with the initial conditions set to $I(0) = 0.3$ and $S(0) = 0.7$. The simulation time is limited to $t_{\rm max} = 100$ since samples with $I(t) = 0$ start to emerge beyond this point due to finite-size effects. We estimate $\alpha = 1.79$ from the scaling plot $I(t)t^\alpha$, which aligns closely with the β estimate. The patterns observed in the plots of $I(t)$ and $I(t)t^\alpha$ vs. t mirror those in Figs. 2 and 4, hence we omit these plots for brevity. Utilizing the values of $\bar{\lambda}_c^{\rm HMF}$ and $\bar{\lambda}_c^{\rm SIS}$ from Eq. (37), we derive $\lambda_c^{\rm th}(h) = 0.03923$ for $h = 0.5$ using Eq. (29). This theoretical value is 2.76% higher than the estimated $\lambda_c = 0.0381$.

For $h = 0.9$, we increase λ by 10^{-4} across the range from 0.0077 to 0.0081. Subsequently, we measure $I(\Delta, t)$ up to $t_{\rm max} = 10^4$ and average I over up to 200 samples, with the initial conditions set to $I(0) = S(0) = 0.5$.

Upon analyzing the data, we discern that $I(\Delta)$ demonstrates the highest linearity at $\lambda_c^* = 0.007235$, with $\beta_{\rm eff}(\Delta)$ remaining stable at approximately 1.9. Fig. 6(c) and 6(d) display the double logarithmic plots of $I(\Delta)$ and $\beta_{\rm eff}(\Delta)$ corresponding to $\lambda_c^* = 0.007235$. Hence, we estimate $\lambda_c = 0.007235$ and $\beta = 1.9051(5)$ using the slope of the least-square fitting line in Fig. 6(c). We conduct simulations with 270 samples, beginning with random initial conditions of $I(0) = 1.0$, and $I(t)$ is measured up to $t_{\rm max} = 700$. We estimate $\alpha = 1.93$ from the scaling plot $I(t)t^\alpha$, which again aligns well with the β estimate. Similar to the $h = 0.5$ case, we derive $\lambda_c^{\rm th}(h) = 0.00762$ for $h = 0.9$ using the values of $\bar{\lambda}_c^{\rm HMF}$ and $\bar{\lambda}_c^{\rm SIS}$ from Eq. (37). This theoretical value is approximately 5% higher than the simulation value of $\lambda_c = 0.007235$.

The estimated values of λ_c for $h = 0.5$ and 0.9 agree well with the predicted values $\lambda^{\rm th}(h)$ from Eqs. (29) and (32), which are presented in Eqs.(14) and (15) of the main text. Additionally, it is worth noting that the estimate of β for $h = 0.9$ is even closer to $\beta^{\rm HMF}$ than the estimate for $h = 0.5$. Here, $\beta^{\rm HMF} = 1/(\gamma - 3) = 2$ for the case of $\gamma = 3.5$. This phenomenon is a result of the SIRS model becoming more akin to the SIS model as the value of h increases, owing to the relationship described in Eq. (24), which is presented as Eq.(12) in the main text. Considering the fact that larger networks are anticipated to yield β values that are closer to $\beta^{\rm HMF}$, we can be more confident in asserting that the simulation results for $\gamma = 3.5$ validate the predictions for β and α that the HMF theory puts forward, i.e. $\beta^{\rm HMF} = 1/(\gamma - 3)$ and $\alpha = \beta^{\rm HMF}$ for $3 < \gamma < 4$.

Through these simulation results, we come to the conclusion that while dynamic correlations do impact the thresholds, they do not exert the same influence on the critical exponents in quenched SFNs.

E. Verification of bond-detailed-balance conditions

We verify the equations of bond-detailed-balance conditions, which are represented by Eqs. (7) through (12), through simulations. Let us define n_s as the count of susceptible nodes, given by $n_s = k - j - n$. This signifies the number of susceptible nodes among the k neighbors for a given combination of j and n. By rearranging Eqs. (7) through (12), we obtain six equalities to be verified, as presented below.

$$\langle \Delta\Pi_{SS} \rangle = 0 : \sum_{kjn} \lambda S_{kjn} j n_s = \sum_{kjn} h R_{kjn} n_s \ ,$$

$$\langle \Delta\Pi_{II} \rangle = 0 : \sum_{kjn} \lambda S_{kjn} j^2 = \sum_{kjn} r I_{kjn} j \ ,$$

$$\langle \Delta\Pi_{RR} \rangle = 0 : \sum_{kjn} r I_{kjn} n = \sum_{kjn} h R_{kjn} n \ ,$$

$$\begin{aligned} \langle \Delta\Pi_{SI} \rangle = 0 \quad : \quad & \sum_{kjn} \lambda S_{kjn} j n_s - \sum_{kjn} r I_{kjn} n_s \\ = \ & \sum_{kjn} \lambda S_{kjn} j^2 - \sum_{kjn} h R_{kjn} j \ , \end{aligned}$$

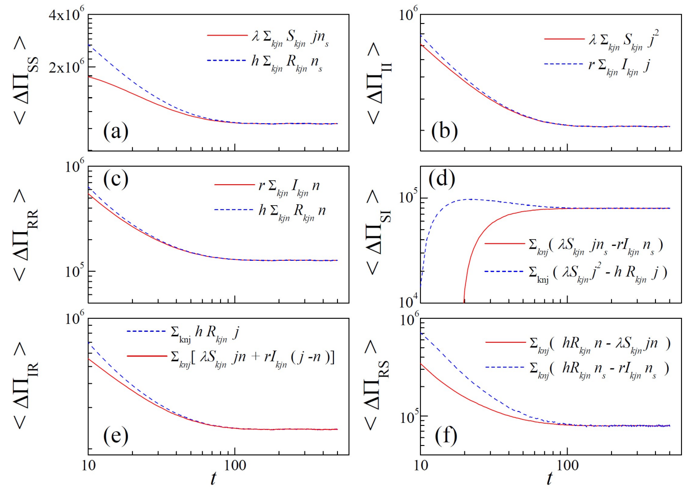

FIG. 7: The verification of $\langle \Delta\Pi_{..} \rangle = 0$ for $h = 0.5$ and $\lambda = 0.087$ ($\Delta = 7.4 \times 10^{-3}$) on the quenched SFN with $\gamma = 5$, $N = 10^7$, $k_{\min} = 5$, and $k_{\max} = 100$: Each panel displays both the left-hand and right-hand sides of the equalities from Eq. (38). These values are presented as solid and dashed lines, respectively, as specified in the legend. The y-axis title indicates the corresponding bond change. Across all panels, it is evident that the values on both sides of each equality align perfectly, thus serving as confirmation of the bond-detailed-balance conditions outlined in Eq. (38).

$$\langle \Delta\Pi_{IR} \rangle = 0 : \sum_{kjn} hR_{kjn}j = \sum_{kjn} \lambda S_{kjn}jn + \sum_{kjn} rI_{kjn}(j-n) \ ,$$

$$\begin{aligned} \langle \Delta\Pi_{RS} \rangle = 0 \quad &: \quad \sum_{kjn} hR_{kjn}n - \sum_{kjn} \lambda S_{kjn}jn \\ &= \sum_{kjn} hR_{kjn}n_s - \sum_{kjn} rI_{kjn}n_s \ . \end{aligned} \tag{38}$$

All terms on both sides of the six equalities are calculated up to $t_{\max} = 500$ and then averaged over 10 samples. The simulations are conducted on the same quenched SFNs with parameters $\gamma = 5$, $N = 10^7$, $k_{\min} = 5$, and $k_{\max} = 100$. Specifically, the simulations are carried out for a case with $\lambda = 0.087$ and $h = 0.5$. The estimated value of λ_c for $h = 0.5$ is $\lambda_c = 0.0796$ (as shown in Fig. 1), making the considered λ to be close to λ_c, leading to $\Delta = \lambda - \lambda_c = 7.4 \times 10^{-3}$.

Fig. 7 presents six plots depicting the left-hand-side and the right-hand-side of each equality from Eq. (38). For instance, Fig. 7(a) displays the comparison $\sum_{kjn} \lambda S_{kjn}jn_s = \sum_{kjn} hR_{kjn}n_s$, where $\langle \Delta\Pi_{SS} \rangle = 0$. As observed, both sides of each equality demonstrate a decay in time and eventually converge to each other within the stationary state, which occurs after around $t \approx 100$.

Furthermore, we have performed additional simulations for cases where λ is significantly different from λ_c, specifically $\lambda = 0.15$ and 0.3. The results of these simulations also show the alignment of both sides of the six equalities in the stationary state, similar to the pattern exhibited in Fig. 7. Therefore, based on the simulation results that encompass scenarios near and far from the threshold, it is convincingly demonstrated that the bond-detailed-balance conditions persist within the stationary state of the endemic phase in the SIRS model.

III. THE CONTACT PROCESS

The contact process (CP) stands as the archetype among nonequilibrium models that belong to the directed percolation universality class, exhibiting the absorbing phase transition on lattice structures [3–5].

In the CP model, individuals are divided into two categories: susceptible (S) and infected (I). The processes of infection and recovery take place in the following manner: (i) A node is randomly selected. Should the chosen node be infected, a neighbor of the selected node is subsequently chosen at random. (ii) If the chosen neighbor is susceptible, it becomes infected with a rate denoted as λ. (iii) The selected node then undergoes recovery at a rate of r. It is worth noting that a key difference between the CP model and the SIS model is that in the CP model, a single neighbor is randomly selected for the infection process, which takes place only if the chosen neighbor is susceptible. This random selection constitutes a crucial characteristic of the CP model, causing its threshold and critical exponents to deviate from those of the SIS model on complex networks [10].

On complex networks, the Contact Process exhibits an absorbing phase transition at a threshold $\bar{\lambda}_c = \lambda_c / r$. In the context of heterogeneous mean-field theory, $\bar{\lambda}_c$ is predicted to be equal to 1 for networks with $\gamma > 2$ [10]. However, the accuracy of HMF theory in predicting the CP model's threshold $\bar{\lambda}_c$ on quenched networks remains uncertain. Similar to the SIR, SIS, and SIRS models, the dynamic correlations between connected nodes are also expected to play a crucial role in determining the CP model's threshold $\bar{\lambda}_c$ on quenched networks. To address this, the Heterogeneous Pair-Approximation (HPA) was introduced for the CP model [11]. The HPA focuses on pairs of nodes connected to each other and formulates rate equations for the density of total pairs consisting of infected-susceptible nodes, as opposed to the rate equations for infected nodes directly. However, the HPA only provides a self-consistent equation for $\bar{\lambda}_c$ in the steady state, and numerical methods are used to determine $\bar{\lambda}_c$ from this equation [11]. The self-consistent equation from the HPA is expressed as follows:

$$\frac{\bar{\lambda}_c}{\langle k \rangle} \sum_k \frac{k(2k-1)}{2k + \bar{\lambda}_c} P(k) = 1, \tag{39}$$

where $P(k)$ represents the degree distribution. The authors of Ref. [11] compared the results of Monte Carlo simulations with the numerically obtained values of $\bar{\lambda}_c$ from Eq. (39) for quenched scale-free networks (SFNs)

with γ values ranging from 2.3 to 3.5, showing a high degree of agreement. The HPA succeeds in capturing the impact of dynamic correlations, although it falls short of providing an explicit expression for the CP model's threshold.

For the Contact Process, the introduction of random selection of one neighbor poses a theoretical challenge in determining $\bar{\lambda}_c$ in comparison to the SIS and SIRS models. Hence, it is crucial to devise an approach that incorporates the dynamic correlations arising from this random selection process, in a manner similar to the utilization of the probability p_k in the SIS and SIRS models.

Additionally, there exists uncertainty regarding whether the CP demonstrates a transition on quenched SFNs when $\gamma < 3$. In cases where $\langle k^2 \rangle$ diverges and a minority of hubs control a significant portion of total links, the network structure is highly irregular. This situation leads to the majority of infections occurring at hubs and their immediate neighbors. This intricate network structure emphasizes the heightened importance of dynamic correlations for quenched SFNs with $\gamma < 3$, rendering the predictions of the HMF theory for $\bar{\lambda}_c$ less reliable.

As is evident in the SIS and SIRS models, the value of $\bar{\lambda}_c$ is influenced by both the first and second moments of node degree. Given this, it is reasonable to anticipate that this dependency is also valid for the CP model. Thus, it becomes imperative to derive an accurate expression for the threshold $\bar{\lambda}_c$ of the CP model on quenched networks to address the challenges outlined above.

To derive an explicit expression for $\bar{\lambda}_c$ in the context of the CP model on quenched networks, our approach involves initially devising a means to represent the dynamic correlations stemming from the random selection process using the previously defined probability p_k in the SIRS model. Subsequently, we apply the methodology outlined in the main text to attain our desired expression.

Similar to the approach employed for the SIRS model in the main text, we introduce the variables S_{kj} and I_{kj} which represent the number of nodes connected to j infected neighbors within the groups of S_k and I_k nodes, respectively. In light of the principle of maximum entropy, we assume uniform values for p_k across all degrees, denoted as $p_k = p\ \forall k$. Taking into account the impact of randomly selecting one neighbor, we can formulate the rate equation for $I_k(t)$ in the CP model through the Heterogeneous Mean-Field approximation as follows:

$$\begin{aligned}
\frac{dI_k}{dt} &= -rI_k + \lambda k S_k \sum_{k'} \frac{1}{k'} \frac{I_{k'}}{N_{k'}} P(k'|k) \\
&= -rI_k + \lambda k S_k \sum_{k'} \frac{1}{k'} \frac{I_{k'}}{N_{k'}} \left(\frac{k'}{\langle k \rangle} P(k') \right) \\
&= -rI_k + \lambda k S_k \frac{1}{\langle k \rangle} \left\langle \frac{I_k}{N_k} \right\rangle , \qquad (40)
\end{aligned}$$

where $1/k'$ in the first line arises due to the stochastic selection of one neighbor from a pool of k' neighbors and the second line makes use of the property $P(k'|k) = k'P(k')/\langle k \rangle$ for uncorrelated networks. This rate equation effectively encapsulates the influence of dynamic correlations, stemming from the random neighbor selection, on the evolution of infected nodes in the CP model.

To incorporate the term p_k into Eq. (40), similar to what was done for the SIRS model, we aim to determine the relationship between p_k and the second term in the final line of Eq. (40). In the stationary state, we arrive at a self-consistent equation for I_k by setting $\frac{dI_k}{dt} = 0$ and employing $S_k + I_k = N_k$:

$$rI_k = \lambda k (N_k - I_k) \frac{1}{\langle k \rangle} \left\langle \frac{I_k}{N_k} \right\rangle , \qquad (41)$$

which yields the expression for I_k:

$$\begin{aligned}
\frac{I_k}{N_k} &= \frac{\lambda k \left\langle \frac{I_k}{N_k} \right\rangle}{r\langle k \rangle + \lambda k \left\langle \frac{I_k}{N_k} \right\rangle} \\
&= \frac{\lambda k}{r\langle k \rangle} \left\langle \frac{I_k}{N_k} \right\rangle \left(1 + \frac{\lambda k}{r\langle k \rangle} \left\langle \frac{I_k}{N_k} \right\rangle \right)^{-1} \\
&\overset{I_k \to 0}{=} \frac{\lambda k}{r\langle k \rangle} \left\langle \frac{I_k}{N_k} \right\rangle , \qquad (42)
\end{aligned}$$

with the last equality valid near $\bar{\lambda}_c$ due to $I_k = 0$ at $\bar{\lambda}_c$. By utilizing the expression for I_k from Eq. (42), the expression for $p = p_k$ can be deduced from the HMF theory:

$$\begin{aligned}
p &= \sum_k \frac{k}{\langle k \rangle} \frac{I_k}{N_k} P(k) \\
&= \sum_k \frac{\lambda k^2}{r\langle k \rangle^2} \left\langle \frac{I_k}{N_k} \right\rangle \left(1 + \frac{\lambda k}{r\langle k \rangle} \left\langle \frac{I_k}{N_k} \right\rangle \right)^{-1} P(k) \\
&\overset{I_k \to 0}{=} \frac{\lambda \langle k^2 \rangle}{r\langle k \rangle^2} \left\langle \frac{I_k}{N_k} \right\rangle . \qquad (43)
\end{aligned}$$

The first equality represents the expression of p as given by the HMF theory for the SIS and SIRS models. On the other hand, the summation in the second line of Eq. (40) can be redefined using the p expression from Eq. (43):

$$\begin{aligned}
&\sum_k \frac{1}{\langle k \rangle} \frac{I_k}{N_k} P(k) \\
&= \sum_k \frac{\lambda k}{r\langle k \rangle^2} \left\langle \frac{I_k}{N_k} \right\rangle \left(1 + \frac{\lambda k}{r\langle k \rangle} \left\langle \frac{I_k}{N_k} \right\rangle \right)^{-1} P(k) \\
&\overset{I_k \to 0}{=} \frac{\lambda \langle k \rangle}{r\langle k \rangle^2} \left\langle \frac{I_k}{N_k} \right\rangle = \frac{\langle k \rangle}{\langle k^2 \rangle} p . \qquad (44)
\end{aligned}$$

This allows us to rewrite the rate equation $\frac{dI_k}{dt}$ from Equation (40) as:

$$\frac{dI_k}{dt} = -rI_k + \lambda k S_k p \frac{\langle k \rangle}{\langle k^2 \rangle} , \qquad (45)$$

TABLE I: The bond changes by all possible events in a time interval dt of the CP on a quenched network. Λ represents the scaled infection rate defined as $\Lambda = \frac{<k>}{<k^2>}\lambda$, and $\Sigma = \sigma + \pi$.

Event	$S_{kj} \to I_{kj}$	$I_{kj} \to S_{kj}$
Probability	$\Lambda S_{kj} j/\Sigma$	rI_{kj}/Σ
$\Delta\Pi_{SS}$	$-(k-j)$	$(k-j)$
$\Delta\Pi_{II}$	j	$-j$
$\Delta\Pi_{SI}$	$-j+(k-j)$	$j-(k-j)$

which achieves our objective of incorporating p. This equation provides the stationary-state relationship:

$$rI_k = \lambda k S_k p \frac{\langle k\rangle}{\langle k^2\rangle} = \lambda k (N_k - I_k) p \frac{\langle k\rangle}{\langle k^2\rangle} \ . \quad (46)$$

The equation for I_k involving p is given by:

$$I_k = \frac{\lambda p k N_k \langle k\rangle}{r\langle k^2\rangle + \lambda p k \langle k\rangle} \ . \quad (47)$$

As λ approaches λ_c, Eq. (45) and (46) become increasingly accurate.

Eq. (45) aligns with the corresponding equations of the SIRS model (Eq. (2) in the main text) and the SIS model of Ref. [12] by introducing a scaled infection rate denoted as Λ. This scaled infection rate, defined as $\Lambda = \lambda\langle k\rangle/\langle k^2\rangle$, allows for a seamless application of our method to the CP model.

The total infection rate (σ) and the total recovery rate (π) are given by:

$$\sigma = \Lambda \sum_{k,j} S_{kj} j \ , \quad \pi = r \sum_{k,j} I_{kj} \ . \quad (48)$$

In Table 1, we summarize all possible changes of the numbers of all types of bonds in time interval dt, along with the corresponding probabilities. From Table I, we deduce three equations based on the bond-detailed-balance conditions. These equations result from summing the product of each contribution to $\Delta\Pi_{ab}$ (where a and b can be either I or S) and the corresponding probability. By setting $\langle\Delta\Pi_{ab}\rangle = 0$, we obtain the following equations:

$$\langle\Delta\Pi_{SS}\rangle = -\sum_{kj} \Lambda S_{kj} j(k-j) + \sum_{kj} rI_{kj}(k-j) = 0, \quad (49)$$

$$\langle\Delta\Pi_{II}\rangle = \sum_{kj} \Lambda S_{kj} j^2 - \sum_{kj} rI_{kj} j = 0 \ , \quad (50)$$

$$\langle\Delta\Pi_{SI}\rangle = \sum_{kj} \Lambda S_{kj} j\Big(-j+(k-j)\Big) + \sum_{kj} rI_{kj}\Big(j-(k-j)\Big) = 0 \ , \quad (51)$$

where $S_{kj} = S_k(1-p_k)^{k-j} p_k^j C_j^k$.

The three equations derived from the bond-detailed-balance conditions can be simplified into a single equation, which corresponds to Eq. (50). To evaluate each term in Eq. (50), we use the identity that the total number of infected neighbors of all susceptible nodes is equal to the total number of susceptible neighbors of all infected nodes, $\Pi_{SI} = \Pi_{IS}$. This leads to another equation:

$$\Pi_{SI} = \Pi_{IS} : \sum_{kj} S_{kj} j = \sum_{kj} I_{kj}(k-j) \ . \quad (52)$$

The second term in Eq. (50) can be obtained from $\sum I_{kj} j = \sum k I_k - \sum S_{kj} j$, utilizing Eq. (52). Using the expressions for S_{kj} and $\bar{p} = 1-p$, we calculate as follows:

$$\begin{aligned}\sum_{kj} S_{kj} j &= \sum_{kj} S_k (1-p)^{k-j} p^j {}_kC_j \cdot j \\ &= \sum_{kj} S_k \bar{p}^{k-j} \left(p\frac{\partial}{\partial p}\right) p^j {}_kC_j \\ &= \sum_k S_k \left(p\frac{\partial}{\partial p}\right)(\bar{p}+p)^k \\ &= \sum_k S_k pk (\bar{p}+p)^{k-1} = \sum_k kpS_k \ . \quad (53)\end{aligned}$$

The first term in Eq. (50) is obtained as:

$$\begin{aligned}\Lambda \sum_{kj} S_{kj} j^2 &= \Lambda \sum_k S_k \left(p\frac{\partial}{\partial p}\right)^2 (\bar{p}+p)^k \\ &= \Lambda \sum_k S_k kp \left[1 + p(k-1)\right] \\ &= r \sum_k I_k \left[1 + p(k-1)\right] \ , \quad (54)\end{aligned}$$

where the relation $rI_k = \Lambda kpS_k$ from Eq. (46) is used in the last line. Combining Eqs. (53) and (54), we can express Eq. (50) as:

$$r\sum_k I_k \left[1+p(k-1)\right] - r\sum_k I_{kj} k + r\sum_k S_k pk = 0. \quad (55)$$

We finally arrive at the equation for I_k by utilizing Eq. (46):

$$\sum_k I_{kj} k - \sum_k \frac{\langle k^2\rangle}{\bar{\lambda}\langle k\rangle} I_k - \sum_k I_k \left[1+p(k-1)\right] = 0 \ . \quad (56)$$

To determine the threshold $\bar{\lambda}_c$ (which equals λ_c/r), we define the left-hand side of Eq. (56) as $f(p)$ and rearrange it as follows:

$$f(p) = \sum_k kI_k(1-p) - \sum_k I_k \left[\frac{\langle k^2\rangle}{\bar{\lambda}\langle k\rangle} + 1 - p\right] \ . \quad (57)$$

The function $f(p)$ is equal to 0 at $p=0$ and becomes negative at $p=1$. As a result, the slope of $f(p)$ at $p=0$ ($df(p)/dp|_{p=0}$) should be positive in order to have a nontrivial solution for $p \in (0,1)$, and it should approach zero as $\bar{\lambda}$ approaches $\bar{\lambda}_c$. Thus, the condition for the

transition to occur is $df(p)/dp|_{p=0} = 0$, similar to the cases of the SIS and SIRS models.

By differentiating $I_k(p)$ of Eq. (47) at $p = 0$, we obtain:

$$\sum_k \frac{dI_k}{dp}\bigg|_{p=0} = \frac{\bar{\lambda} N \langle k \rangle^2}{\langle k^2 \rangle}, \quad \sum_k k \frac{dI_k}{dp}\bigg|_{p=0} = \frac{\bar{\lambda} N \langle k^2 \rangle \langle k \rangle}{\langle k^2 \rangle} . \tag{58}$$

With these expressions, we can derive the equation for $\bar{\lambda}_c$ from $df(p)/dp|_{p=0}$:

$$1 - \frac{\langle k \rangle}{\langle k^2 \rangle} \left[\frac{\langle k^2 \rangle}{\bar{\lambda}_c \langle k \rangle} + 1 \right] = 0 . \tag{59}$$

Solving Eq. (59), we finally obtain the threshold $\bar{\lambda}_c$ for the CP model:

$$\bar{\lambda}_c = \frac{\langle k^2 \rangle}{\langle k^2 \rangle - \langle k \rangle} . \tag{60}$$

Unlike the SIS and SIRS models, the threshold of the CP model remains finite for $\gamma > 2$, even in cases where $\langle k^2 \rangle$ diverges. For $2 < \gamma < 3$, $\langle k^2 \rangle$ diverges as the network size N approaches infinity, leading to $\bar{\lambda}_c = 1$ for $N \to \infty$. While the HMF theory for the CP predicts $\bar{\lambda}_c = 1$ for scale-free networks with $\gamma > 2$ [10], our result aligns with $\bar{\lambda}_c = 1$ only for $2 < \gamma < 3$. On quenched SFNs with $\gamma > 3$ and random networks following a Poissonian degree distribution $P(k)$, $\bar{\lambda}_c$ exceeds 1 and varies with both $\langle k \rangle$ and $\langle k^2 \rangle$.

Furthermore, the threshold $\bar{\lambda}_c$ of the CP model on a regular network with a degree of k_0 and a degree distribution of $P(k) = \delta_{k,k_0}$ is known to be [12]:

$$\bar{\lambda}_c = \frac{k_0}{k_0 - 1} . \tag{61}$$

When substituting $\langle k^2 \rangle = k_0^2$ and $\langle k \rangle = k_0$ for the regular network into Eq. (60), we correctly reproduce the threshold of the CP model on regular networks.

IV. SIMULATION RESULTS OF THE CP ON QUENCHED SCALE-FREE NETWORKS

In the derivation of the threshold of the CP model, a crucial approximation is the representation of dynamic correlations arising from the random selection of one neighbor by $\lambda p \langle k \rangle / \langle k^2 \rangle$ in Eq. (45), which corresponds to scaling λ with $\langle k \rangle / \langle k^2 \rangle$ as $\Lambda = \lambda \langle k \rangle / \langle k^2 \rangle$. Subsequently, we obtain an equation for $dI_k(t)/dt$ that is analogous to the SIS model of Ref. [12] and the SIRS models discussed in the main text. This simplifies the subsequent calculations. To validate the accuracy of this approximation, it's essential to verify one of the bond-detailed-balance conditions, specifically $\Delta \Pi_{II} = 0$ of Eq. (50), i.e., $\bar{\Lambda} \sum_{k,j} k j^2 S_{kj} = \sum_{k,j} j I_{kj}$, through simulations. Here, $\bar{\Lambda} = \bar{\lambda} \langle k \rangle / \langle k^2 \rangle$.

On the quenched SFN with $\gamma = 5$, $N = 10^7$, $k_{\min} = 5$, and $k_{\max} = 100$, where $\langle k \rangle = 6.0942$ and

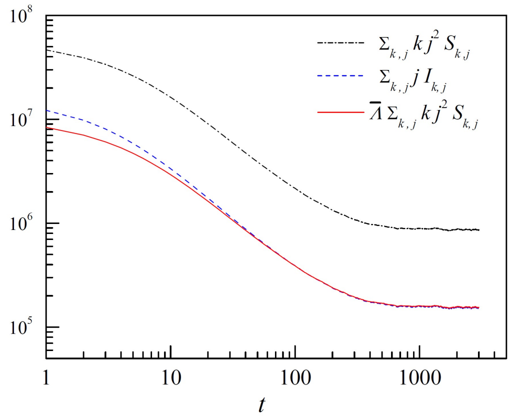

FIG. 8: The quenched SFN of $\gamma = 5$, $N = 10^7$, $k_{min} = 5$, and $k_{max} = 100$, $\bar{\lambda}_c = 1.2091$: Plot at $\bar{\lambda} = 1.225$ ($\Delta = 0.0159$) of the temporal changes of $\sum_{k,j} k j^2 S_{kj}$, $\sum_{k,j} j I_{kj}$, and $\bar{\Lambda} \sum_{k,j} k j^2 S_{kj}$ of Eq. (50), where $\bar{\Lambda} = \bar{\lambda} \langle k \rangle / \langle k^2 \rangle$. Each summation corresponds to the line indicated in the legend. As depicted in the figure, $\bar{\Lambda} \sum_{k,j} k j^2 S_{kj}$ aligns with $\sum_{k,j} j I_{kj}$ in the steady state after approximately $t = 10^3$.

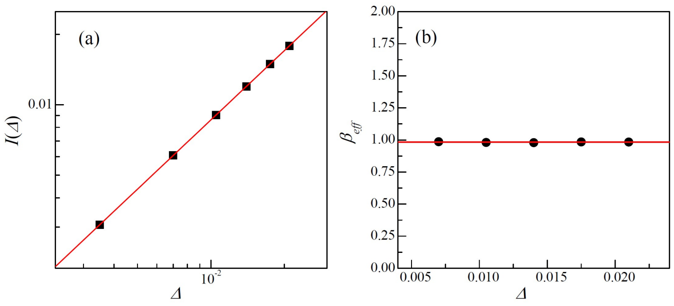

FIG. 9: $I(\Delta)$ for $\gamma = 5$ of the CP: Plots of $I(\Delta)$ and $\beta_{\rm eff}$ vs. $\Delta = \bar{\lambda} - \bar{\lambda}_c$ with $\bar{\lambda}_c = 1.2091$. (a) Each symbol corresponds to $I(\Delta)$ for $\bar{\lambda} = 1.2126$ to 1.2301 increased by 3.5×10^{-3}. The straight line represents the fitting line with a slope of $\beta = 0.9827(7)$. (b) $\beta_{\rm eff}$ is stable and remains close to 1. The horizontal line indicates the slope of the fitting line.

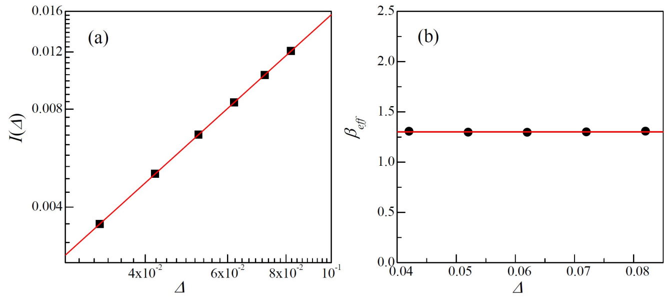

FIG. 10: $I(\Delta)$ for $\gamma = 2.5$ of the CP: Plots of $I(\Delta)$ and $\beta_{\rm eff}$ vs. $\Delta = \bar{\lambda} - \bar{\lambda}_c$ with $\bar{\lambda}_c = 1.078$. (a) Each symbol corresponds to $I(\Delta)$ for $\bar{\lambda} = 1.1$ to 1.16 increased by 0.01. The straight line represents the fitting line with a slope of $\beta = 1.3014(8)$. (b) $\beta_{\rm eff}$ is stable. The horizontal line indicates the slope of the fitting line.

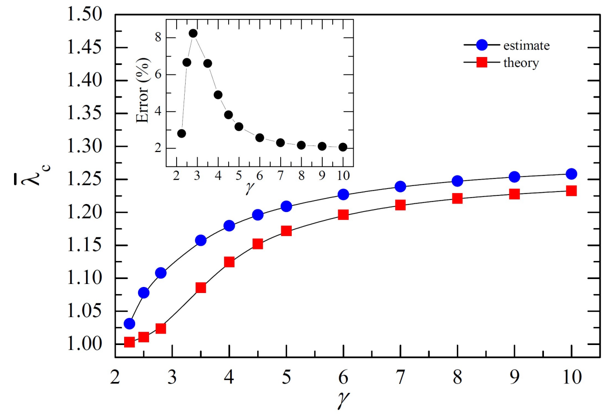

FIG. 11: Phase diagram of the CP on quenched SFNs of $N = 10^7$ and $k_{min} = 5$: Plots of $\bar{\lambda}_c$ vs. γ. The circles and squares represent the estimated values of $\bar{\lambda}_c$ from simulations and the theoretical $\bar{\lambda}_c$ obtained from Eq. (60), respectively, for SFNs with various γ values ranging from 2.25 to 10. The inset displays the errors in the estimates compared to the theoretical values. The solid line connecting the symbols serves as a visual aid.

$\langle k^2 \rangle = 41.547$, Monte Carlo simulations of the CP model are conducted starting with random initial conditions of $I(0) = 0.5$. The estimate of $\bar{\lambda}_c (= \lambda_c/r)$ is determined as $\bar{\lambda}_c = 1.2091$. (Further details regarding the estimation of $\bar{\lambda}_c$ are provided below.) At $\bar{\lambda} = 1.225$ ($\Delta = \bar{\lambda} - \bar{\lambda}_c = 0.0159$), both $\sum_{k,j} kj^2 S_{kj}$ and $\sum_{k,j} jI_{kj}$ are measured up to $t = 3 \times 10^3$ and averaged over 5 different initial distributions of $I(0) = 0.5$. Figure 8 illustrates $\sum_{k,j} kj^2 S_{kj}$, $\sum_{k,j} jI_{kj}$, and $\bar{\Lambda} \sum_{k,j} kj^2 S_{kj}$, thereby confirming $\bar{\Lambda} \sum_{k,j} kj^2 S_{kj} = \sum_{k,j} jI_{kj}$ in the steady state, as anticipated when $\bar{\lambda} \to \bar{\lambda}_c$.

To verify the threshold $\bar{\lambda}_c$ as given by Eq. (60) (referred to as Eq. (16) in the main text), we conducted simulations on quenched SFNs with varying values of γ ranging from 2.25 to 10, and N fixed at 10^7, along with $k_{\min} = 5$. The upper limit of the degree, $k_{\max}$, increases from 27 for $\gamma = 10$ to 5×10^3 for $\gamma = 2.25$.

We estimated $\bar{\lambda}_c$ for each value of γ using methods similar to those used for the SIRS model. As examples, simulation results for $\gamma = 5$ and 2.5 are depicted in Figs .9 and 10. At the estimated $\bar{\lambda}_c$, we confirmed the power-law decay of $I(t)$ with $\alpha = \beta$. For the CP model on quenched SFNs, the exponent β^{HMF} follows $\beta^{\text{HMF}} = 1$ for $\gamma > 3$ and $\beta^{\text{HMF}} = 1/(\gamma - 2)$ for $2 < \gamma < 3$, yielding $\beta^{\text{HMF}} = 2$ for $\gamma = 2.5$ and $\beta^{\text{HMF}} = 1$ for $\gamma = 5$ [9–11]. Comparing β^{HMF} with our estimates, we find excellent agreement for $\gamma = 5$, but discrepancies for $\gamma = 2.5$ as evident in Figs. 9 and 10. Similarly, we also observe underestimation of β for $\gamma = 2.25$ and 2.8. The measurement of scaling exponents often encounters substantial finite-size effects for $\gamma < 3$, contributing to the underestimation of β in this range [9, 10]. By estimating $\bar{\lambda}_c$ while incrementing γ from 2.25 to 10, we map out the phase diagram illustrated in Fig. 11. In this phase diagram, each theoretical $\bar{\lambda}_c$ is computed using the $\langle k \rangle$ and $\langle k^2 \rangle$ specific to the corresponding network, and plugged into Eq. (60). The errors in the estimates are presented as percentages, calculated using the formula $100 \times (\text{estimate} - \bar{\lambda}_c)/\bar{\lambda}_c$. As evident in the inset of Figure 11, errors sharply increase near $\gamma = 3$, reflecting the significant structural change of the network at this point. For other values of γ, errors remain quite small, ranging from 2% to 4%. Thus, the simulation outcomes robustly endorse the theoretical threshold of Eq. (60) and the validity of our approach for the CP model.

---

[1] Noh, J. D. and Park, H. Critical behavior of the contact process in annealed scale-free networks. *Phys. Rev. E* **79**, 056115 (2009).

[2] Kwon, S. and Kim, Y. Epidemic spreading in annealed directed networks: Susceptible-infected-susceptible model and contact process. *Phys. Rev. E* **87**, 012813 (2013).

[3] Marro, J. and Dickman, R. *Nonequilibrium Phase Transitions in Lattice Models* (Cambridge University Press, 1999).

[4] Hinrichsen, H. Non-equilibrium critical phenomena and phase transitions into absorbing states. *Adv. Phys.* **49**, 815-958 (2010).

[5] Henkel, M., Hinrichsen, H. and Lübeck, S. *Non-Equilibrium Phase Transitions* Vol. I: Absorbing Phase Transitions (Springer, 2009).

[6] Pastor-Satorras, R. and Vespignani, A. Epidemic Spreading in Scale-Free Networks. *Phys. Rev. Lett.* **86**, 3200-3203 (2001).

[7] Pastor-Satorras, R. and Vespignani, A. Epidemic dynamics and endemic states in complex networks. *Phys. Rev. E* **63**, 066117 (2001).

[8] Pastor-Satorras, R., Castellano, C., Van Mieghem, P. and Vespignani, A. Epidemic processes in complex networks. *Rev. Mod. phys.* **87**, 925-979 (2015).

[9] Hong, H., Ha, M. and Park, H. Finite-Size Scaling in Complex Networks. *Phys. Rev. Lett.* **98**, 258701 (2007).

[10] Castellano, C. and Pastor-Satorras, R. Non-Mean-Field Behavior of the Contact Process on Scale-Free Networks. *Phys. Rev. Lett.* **96**, 038701 (2006).

[11] Mata, A. S., Ferreira, R. S. and Ferreira, S. C. Heterogeneous pair-approximation for the contact process on complex networks. *New J. Phys.* **16** 053006 (2014).

[12] Cai, C.-R., Wu, Z.-X., Chen, M. Z. Q., Holme, P. and Guan, J.-Y. Solving the Dynamic Correlation Problem of the Susceptible-Infected-Susceptible Model on Networks. *Phys. Rev. Lett.* **116**, 258301 (2016).
